# Microelimination of hepatitis C in dialysis units: results from the Brazilian Registry for the elimination of HCV

**DOI:** 10.1590/2175-8239-JBN-2026-0092en

**Published:** 2026-09-14

**Authors:** Patrícia Ferreira Abreu, Pablo Rodrigues Costa Alves, Paulo Lisboa Bittencourt, Isadora Cartaxo de Sousa Calvo, Osvaldo Merege Vieira, Marcelo Mazza do Nascimento, Felipe Costa Neves, Ana Flavia Moura, Daniel Costa Chalabi Calazans, Juan Miguel Villalobos Salcedo, Cristina Melo Rocha, Cirley Maria de Oliveira Lobato, Alex Vianey Callado França, Alessandra Maria Mont’Alverne Pierre, Adalgisa de Souza Paiva Ferreira, Edmundo Pessoa Lopes, Jose Eymard Moraes de Medeiros, Francisco José Dutra Souto, Rodrigo Sebba Aires, Liliana Sampaio Costa Mendes, Silvia Naomi de Oliveira Uehara, Luciana Lofego Gonçalves, Lilian Pires de Freitas do Carmo, Cláudia Alexandra Pontes Ivantes, Hugo Cheinquer, Tatiara Bueno Parreira, Karla Cristina Silva Petruccelli Israel, Verônica de Jesus Rodrigues Cardozo Costa, Hivis da Costa Sousa, Jarinne Camilo Landim Nasserala, Kleyton de Andrade Bastos, Maria Eliete Pinheiro, Artur Quintiliano Bezerra da Silva, Claudia Maria Costa de Oliveira, Flávio Henrique Soares Barros, Marclébio Manuel Coêlho Dourado, Ciro Bruno Costa, Ricardo Araújo Mothé, Alexandre Silvestre Cabral, Denise Rodrigues Simão, Fellype de Carvalho Barreto, Paulo Henrique Fraxino, Natasha Silva Constancio, Dirceu Reis da Silva, Álvaro Pacheco Silva, Lúcio Roberto Requião Moura, David Jose de Barros Machado, Alexandre Minetto Brabo, Alice Pignaton Naseri, Tomás Ribeiro Carvalho, Nathalia da Fonseca Pestana, Pedro Tulio Monteiro de Castro e Abreu Rocha, Leila Maria Soares Tojal de Barros Lima, Renata M. Perez, Janaina Luz Narciso-Schiavon, Antônio Ricardo Cardia Ferraz de Andrade, Bruno Zawadzki, José A. Moura-Neto, Maria Lucia Gomes Ferraz

**Affiliations:** 1 Sociedade Brasileira de Nefrologia, São Paulo, SP, Brazil.; 2 Universidade Federal de São Paulo, São Paulo, SP, Brazil.; 3 Universidade Federal da Paraíba, João Pessoa, PB, Brazil.; 4 Centro Municipal de Nefrologia de João Pessoa, João Pessoa, PB, Brazil.; 5 Sociedade Brasileira de Hepatologia, São Paulo, SP, Brazil.; 6 Instituto Brasileiro do Fígado, São Paulo, SP, Brazil.; 7 Escola Bahiana de Medicina e Saúde Pública, Salvador, BA, Brazil.; 8 Hospital Português, Salvador, BA, Brazil.; 9 Universidade de São Paulo, Faculdade de Medicina de Ribeirão Preto, Ribeirão Preto, SP, Brazil.; 10 Universidade Federal do Paraná, Curitiba, PR, Brazil.; 11 Universidade Federal de Rondônia, Porto Velho, RO, Brazil.; 12 Universidade do Estado do Amazonas, Manaus, AM, Brazil.; 13 Universidade Federal do Acre, Rio Branco, AC, Brazil.; 14 Secretaria Estadual de Saúde do Acre, Rio Branco, AC, Brazil.; 15 Universidade Federal de Sergipe, Aracaju, SE, Brazil.; 16 Hospital Geral de Fortaleza, Fortaleza, CE, Brazil.; 17 Universidade Federal do Maranhão, São Luís, MA, Brazil.; 18 Universidade Federal de Pernambuco, Hospital das Clínicas, Recife, PE, Brazil.; 19 Universidade Federal de Pernambuco, Recife, PE, Brazil.; 20 Universidade Federal de Mato Grosso, Cuiabá, MT, Brazil.; 21 Universidade Federal de Goiás, Goiânia, GO, Brazil.; 22 Hospital de Base do Distrito Federal, Brasília, DF, Brazil.; 23 Universidade Federal de Mato Grosso do Sul, Campo Grande, MS, Brazil.; 24 Universidade Federal do Espírito Santo, Vitória, ES, Brazil.; 25 Universidade Federal de Minas Gerais, Belo Horizonte, MG, Brazil.; 26 Hospital de Clínicas de Porto Alegre, Porto Alegre, RS, Brazil.; 27 Universidade Federal do Amazonas, Manaus, AM, Brazil.; 28 Universidade Federal do Amapá, Hospital Universitário, Macapá, AP, Brazil.; 29 Hospital do Rim do Acre, Rio Branco, AC, Brazil.; 30 Universidade Federal de Alagoas, Maceió, AL, Brazil.; 31 Universidade Federal do Rio Grande do Norte, Natal, RN, Brazil.; 32 Universidade Federal do Ceará, Hospital Universitário Walter Cantídio, Fortaleza, CE, Brazil.; 33 Hospital Santa Isabel, Blumenau, SC, Brazil.; 34 Conselho Regional de Medicina do Paraná, Câmara Técnica de Nefrologia, Curitiba, PR, Brazil.; 35 Associação Renal Vida, Blumenau, SC, Brazil.; 36 Universidade de São Paulo, Faculdade de Medicina, Hospital das Clínicas, São Paulo, SP, Brazil.; 37 Hospital Alemão Oswaldo Cruz, São Paulo, SP, Brazil.; 38 Rede D’Or, Regional ABC, Santo André, SP, Brazil.; 39 Hospital de Base de Bauru, Bauru, SP, Brazil.; 40 Secretaria Estadual de Saúde do Espírito Santo, Vitória, ES, Brazil.; 41 Universidade Federal do Espírito Santo, Hospital Universitário Cassiano Antonio Moraes, Vitória, ES, Brazil.; 42 Santa Casa de Misericórdia de Passos, Passos, MG, Brazil.; 43 Universidade Federal do Rio de Janeiro, Hospital Universitário Clementino Fraga Filho, Rio de Janeiro, RJ, Brazil.; 44 Universidade Federal do Rio de Janeiro, Rio de Janeiro, RJ, Brazil.; 45 Universidade Federal de Santa Catarina, Florianópolis, SC, Brazil.; 46 Universidade Federal da Bahia, Salvador, BA, Brazil.; 47 DaVita Tratamento Renal, Rio de Janeiro, RJ, Brazil.

**Keywords:** Hepatitis C, Hemodialysis, Peritoneal Dialysis, Renal Dialysis, Renal Insufficiency, Chronic, Antiviral Agents, Sustained Virologic Response

## Abstract

**Introduction:**

Hepatitis C virus (HCV) infection remains a major public health challenge, particularly among patients undergoing dialysis, who are disproportionately vulnerable and at increased risk of transmission. Micro-elimination strategies targeting high-risk subpopulations are a critical component for achieving the World Health Organization global HCV elimination goals.

**Methods:**

This national, multicenter, prospective implementation study was conducted within the framework of the Brazilian Registry for the Elimination of HCV in Dialysis Units. All 904 active dialysis units in the country were invited; 384 (42.5%) joined voluntarily, and, within each, all eligible patients were enrolled (census) between 2021 and 2024. The HCV cascade of care was evaluated, including serological screening (anti-HCV), virological confirmation (HCV-RNA), treatment with direct-acting antivirals (DAAs), and assessment of sustained virologic response (SVR).

**Results:**

A total of 66,601 patients were evaluated, yielding a national anti-HCV prevalence of 2.08%. HCV-RNA testing coverage reached 65.4%, with a positivity rate of 64.2% among those tested, resulting in an adjusted prevalence of active viremia of 1.34%. Among patients with detectable viremia, 74.2% initiated antiviral therapy, 92.6% completed treatment, and 93.3% achieved SVR. The main barriers identified along the cascade of care included limited availability of diagnostic tests and patient mortality.

**Discussion:**

These findings indicate that Brazil has achieved robust therapeutic effectiveness indicators, placing the country at an advanced stage toward HCV elimination within the dialysis population. Nonetheless, important operational gaps persist, particularly in diagnostic confirmation.

**Conclusion:**

Elimination of HCV in dialysis units is an achievable goal in Brazil. Sustained success depends on maintaining universal screening, ensuring prompt treatment initiation for confirmed cases, and strengthening continuous integration between services.

## INTRODUCTION

Hepatitis C virus (HCV) infection remains one of the major global public health challenges, despite the therapeutic advances achieved with direct-acting antivirals (DAAs), which yield cure rates exceeding 95%. It is estimated that approximately 58 million people are living with chronic HCV infection and that 1.5 million new cases occur each year worldwide^1,2^. According to the World Health Organization (WHO) and international guidelines, such as those from Kidney Disease: Improving Global Outcomes (KDIGO) and the European Association for the Study of the Liver (EASL), eliminating hepatitis C by 2030 depends on integrated strategies encompassing universal screening, diagnostic confirmation, timely access to treatment, and monitoring of virologic response after therapy.

In this context, the concept of micro-elimination—an approach focused on targeted treatment of high-risk subgroups—has been shown to be an effective strategy for achieving the global goal of HCV elimination. Among high-risk groups, patients receiving kidney replacement therapy, particularly those on hemodialysis, are especially notable. In this population, vulnerability arises from multiple factors: frequent vascular access manipulation, a shared-care environment, potential exposure to blood and blood products, and an altered immune status. In addition, transmission outbreaks in dialysis services continue to be reported, generally related to failures in biosafety measures. The clinical impact is substantial: individuals on dialysis infected with HCV experience poorer quality of life, higher hospitalization rates, higher mortality, and an increased risk of progression to cirrhosis and hepatocellular carcinoma^3,4,5^.

International experience indicates that eliminating HCV in dialysis units is an achievable goal. The multicenter FORMOSA study, conducted in Taiwan, showed that the implementation of universal screening combined with DAA treatment reduced the viremia rate from 7.7% in 2019 to only 0.6% in 2021. These findings support the concept of “treatment as prevention,” that is, treating all patients with active infection as a strategy for micro-elimination and for preventing new cases within dialysis units^6^. According to the Brazilian Dialysis Census of the Brazilian Society of Nephrology, the prevalence of anti-HCV antibodies (anti-HCV) in dialysis units has declined markedly over the past two decades, decreasing from 19.3% in 2000 to 2.1% in 2023—corresponding to approximately 3,300 patients^7^. Nevertheless, this rate remains three times higher than the prevalence observed in the general Brazilian population (0.7%). A systematic review with meta-analysis by Niquini *et al.* documented this decline in Brazil, while cautioning that pooled prevalence estimates of HCV in hemodialysis are sensitive to study size and to selection and publication biases^8^. Importantly, a positive anti-HCV test indicates prior exposure to the virus; however, national data are still lacking regarding the proportion of such individuals with active infection, that is, with detectable viral load.

Eliminating HCV among dialysis patients in Brazil is a feasible goal, particularly given the universal availability of DAAs through the Unified Health System (SUS). To achieve this goal, three pillars must be strengthened: (1) early diagnosis and systematic screening; (2) universal and immediate treatment of detected cases; and (3) rigorous prevention of transmission within dialysis units. Integration among nephrologists, hepatologists, medical societies, and public health managers will be essential to achieve the objectives established by the World Health Organization (WHO), which aims to reduce the incidence of new infections by 80% and HCV-related mortality by 65% by 2030^3,9^. In this context, the Brazilian Registry for the Elimination of Hepatitis C in Dialysis Units was developed as a multicenter, collaborative initiative organized by the Brazilian Society of Nephrology (SBN), in partnership with the Brazilian Society of Hepatology (SBH) and the Brazilian Liver Institute (IBRAFIG), with the aim of identifying, treating, and monitoring virologic response in individuals receiving dialysis treatment in Brazil^10^.

## METHODS

### Study design

This was an observational, multicenter, prospective, registry-based study using real-world data from dialysis units participating in the Brazilian Registry for the Elimination of Hepatitis C in Dialysis Units. The Registry was conceived as a public health initiative to screen, diagnose, treat, and monitor the virologic response of patients with chronic hepatitis C on dialysis, as well as to identify barriers and opportunities along the care pathway. The project was conducted by the Brazilian Society of Nephrology (SBN), in partnership with the Brazilian Society of Hepatology (SBH) and the Brazilian Liver Institute (IBRAFIG). The SBN, SBH, and IBRAFIG appointed, in each federative unit, state representatives— one nephrologist and one hepatologist—responsible for inviting dialysis units of the state, providing training, and overseeing data collection. All participating units had access to a training course on screening, diagnosis, and treatment of hepatitis C in dialysis, in accordance with the Clinical Protocol and Therapeutic Guidelines of the Ministry of Health^9^.

### Population

All 904 active and registered dialysis units in Brazil were invited to participate. Participation was voluntary and conditional on agreement with the standardized screening, diagnostic confirmation, and treatment protocol, in accordance with the clinical protocol of the Ministry of Health^9^. The invitation was universal, and, within each participating unit, all eligible patients were enrolled without subsampling.

All patients on hemodialysis or peritoneal dialysis, regardless of dialysis duration or the etiology of chronic kidney disease, who were under care at the participating units during the study period were eligible. Within each participating unit, all eligible patients were invited to participate and to sign the Informed Consent Form; only those who declined participation were excluded.

### Procedures and data sources

Care was structured into four sequential steps, according to the Clinical Protocol and Therapeutic Guidelines for Hepatitis C of the Ministry of Health^9^: (1) serologic screening—identification of anti-HCV–positive patients, performed as part of routine dialysis-unit practice; (2) virologic confirmation—molecular HCV-RNA testing in all anti-HCV–positive patients; (3) antiviral treatment—offering and implementing DAAs to all HCV-RNA–positive patients; and (4) SVR assessment—monitoring viral load ≥12 weeks after the end of treatment.

Data collection took place between March 2021 and July 2024. Collection followed two phases. In the first, units registered with the SBN and entered patients’ demographic and clinical data, dialysis modality, and anti-HCV result into the project’s electronic platform (https://pesquisa.ibrafig.org.br/). In the second phase, information was consolidated based on reports from the nephrologists and hepatologists designated in each state or retrieved by the data-management team using standardized spreadsheets containing (1) HCV-RNA result; (2) reason for not performing HCV-RNA testing; (3) treatment initiation; (4) treatment completion; (5) SVR occurrence; and (6) reasons for non-treatment among HCV-RNA–positive patients.

### Outcomes and operational definitions

Prior exposure to HCV was defined as the presence of detectable anti-HCV antibodies on serologic testing. Active viremia was defined as detectable HCV-RNA by reverse transcription followed by polymerase chain reaction (RT-PCR). Treatment completion was defined as completion of the prescribed direct-acting antiviral (DAA) regimen, with or without subsequent assessment. Virologic cure, or sustained virologic response (SVR), was defined as undetectable HCV-RNA ≥12 weeks after the end of treatment.

The primary outcome was the prevalence of active viremia in the dialysis population. Secondary outcomes included anti-HCV prevalence, HCV-RNA coverage and positivity, the cascade-of-care indicators (treatment initiation, completion, and SVR), and the reasons for not performing HCV-RNA testing and for non-treatment.

### Statistical analysis

Categorical variables were described as absolute and relative frequencies and compared using Pearson’s chi-square test or Fisher’s exact test when ≥20% of cells had an expected frequency <5. Continuous variables were described as median (interquartile range, IQR) after assessment of the distribution by the Shapiro–Wilk test and compared using the Mann–Whitney test.

The following measures were analyzed: (1) number of participants per stratum; (2) anti-HCV prevalence (n/N); (3) HCV-RNA coverage among anti-HCV–positive individuals (proportion who underwent HCV-RNA testing); (4) HCV-RNA positivity among individuals with known results (HCV-RNA positive divided by the sum of positives and negatives, excluding cases without a result); and (5) viremia prevalence in the total population, presented in two complementary ways: (5a) confirmed (observed), defined as HCV-RNA positive divided by the total number of participants; and (5b) adjusted for incomplete verification under the missing-at-random (MAR) assumption, estimated as the product of anti-HCV prevalence and HCV-RNA positivity among those tested. In addition, the following were described: (6) the cascade of care (anti-HCV positive, HCV-RNA testing performed, HCV-RNA positive, treated, treatment completed, and SVR), with the proportions of each transition; (7) the reasons for not performing HCV-RNA testing among anti-HCV–positive individuals; and (8) the reasons for non-treatment among HCV-RNA–positive individuals. The absence of a result at any step was treated as an outcome of interest and explicitly quantified (see statistical analysis), rather than motivating exclusion.

Prevalences (anti-HCV and confirmed viremia), coverage, and HCV-RNA positivity were accompanied by binomial Wilson confidence intervals; adjusted viremia (MAR) had its interval estimated by the delta method for the product of two independent proportions. The Wilson interval was chosen over the Wald interval because of its better coverage performance for proportions close to zero, a frequent situation in the state-level viremia estimates. The variance was obtained as Var(p_1_ · p_2_) ≈ p_2_^2^ · Var(p_1_) + p_1_^2^ · Var(p_2_). To minimize denominator ambiguity, HCV-RNA positivity included only individuals with known results; coverage used the total number of anti-HCV–positive individuals as the denominator; and, in the cascade, each proportion was calculated relative to the immediately preceding step (treated among HCV-RNA positives, completed among treated, and SVR among completed). Given the large sample size, the national estimates were reported with 99% confidence intervals, and the stratum-level estimates display the raw denominators (n/N), allowing assessment of precision where n is small. All analyses were performed for the national dataset and replicated by region and by federative unit.

To address the incomplete verification of HCV-RNA, three complementary strategies were adopted, without exclusion of cases. First, viremia prevalence was reported as both confirmed (lower bound, which implicitly assumes that all untested patients would be negative) and adjusted under the MAR assumption (which assumes that positivity among the untested equals that observed among the tested individuals, conditional on the stratum). Second, an extreme-scenario (*tipping-point*) sensitivity analysis was performed, assigning a negative or positive result to all untested patients so as to delimit the deterministic bounds of prevalence. Third, to assess the plausibility of the MAR assumption, the characteristics of anti-HCV–positive patients with and without an HCV-RNA result were compared (age, sex, dialysis modality, and etiology of chronic kidney disease), and the homogeneity of HCV-RNA coverage across regions was tested, distinguishing the magnitude of the missing data from their potential informative character. Multiple imputation of HCV-RNA status was not used, as it concerns the confirmation outcome itself, whose imputation would depend on an unverifiable model.

*P* values are two-sided, with α = 0.05, interpreted together with the estimates and their confidence intervals, and not as a dichotomous decision threshold. Analyses were performed using Jamovi 2.6.45 and R 4.3.1.

### Ethical considerations and approval by the research ethics committee

The project was approved by the Research Ethics Committee of the *Universidade Federal de São Paulo* (UNIFESP), under approval number 4,412,987 (CAAE 39200420.2.1001.5505), issued on November 21, 2020.

All participants signed the Informed Consent Form (ICF), in accordance with National Health Council Resolution No. 466/12. The study followed the ethical principles of the Declaration of Helsinki and the current regulations of the National Health Council.

## RESULTS

**Figure 1:**
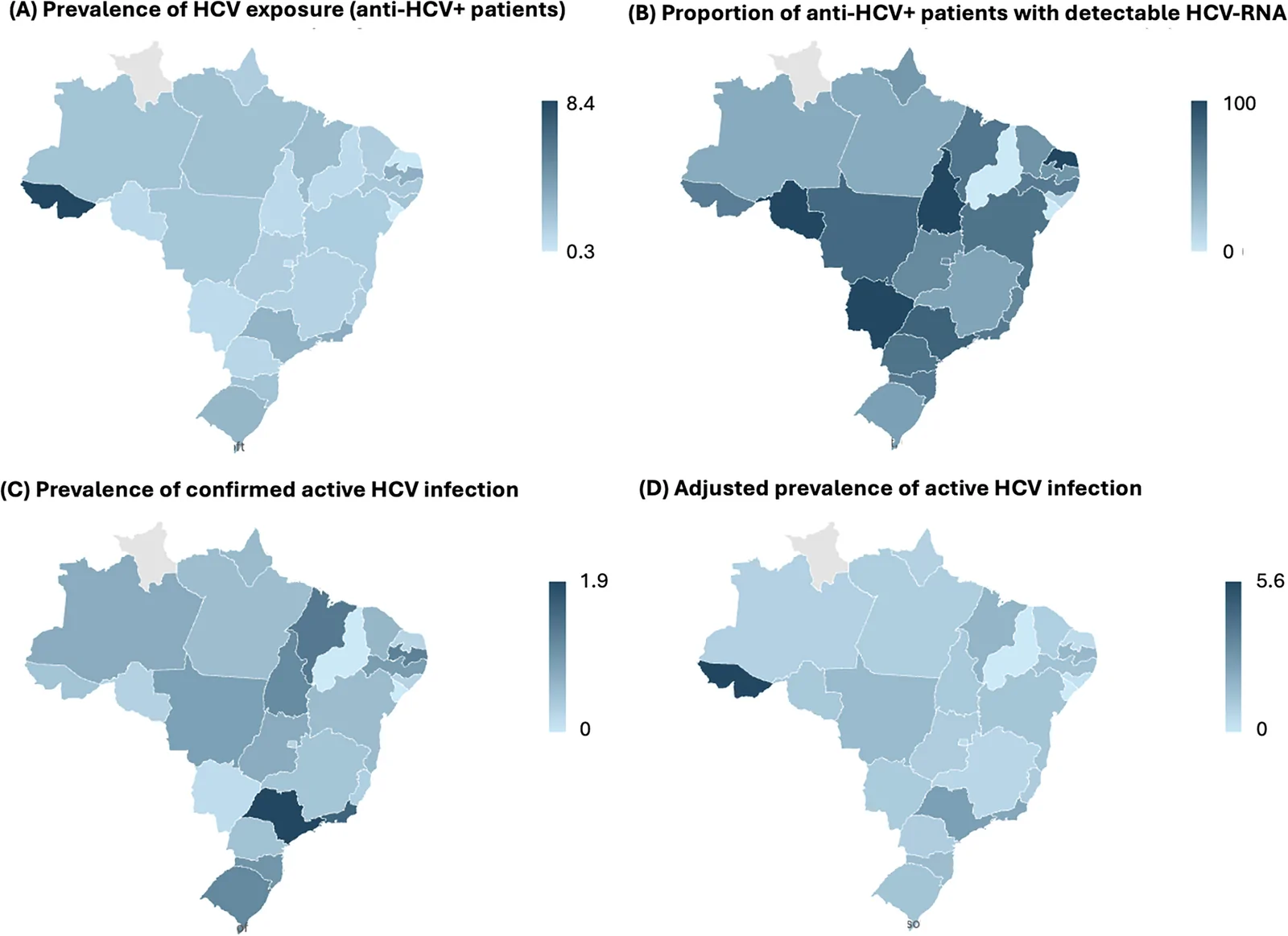
Observed prevalences by federative unit: (A) Prevalence of HCV exposure (anti-HCV–positive patients); (B) Proportion of anti-HCV–positive patients with detectable HCV-RNA; (C) Prevalence of confirmed active HCV infection; (D) Adjusted prevalence of active HCV infection.

A total of 66,601 individuals receiving dialysis (63,028 on hemodialysis and 3,573 on peritoneal dialysis) were evaluated from 384 services across 26 Brazilian states. These 384 services corresponded to 42.5% of the 904 active dialysis units invited. Participation was unevenly distributed across the territory: North, with 27 centers (7.0%) and 3,609 patients; Northeast, with 98 (25.5%) and 20,768; Central-West, with 66 (17.2%) and 8,983; Southeast, with 128 (33.3%) and 24,367; and South, with 65 (16.9%) and 8,874. The distribution of participants relative to the national dialysis network is presented in Figure S1. The national prevalence of anti-HCV positivity was 2.08% (1,385/66,601; 99% CI 1.94–2.23). When stratified by modality, prevalence was 2.17% in hemodialysis (1,366/63,028; 99% CI 2.02–2.32) and 0.53% in peritoneal dialysis (19/3,573; 99% CI 0.30–0.95). At the regional level, anti-HCV positivity estimates indicated geographic heterogeneity: North 2.83% (99% CI 2.20–3.63), Northeast 1.82% (99% CI 1.60–2.08), Central-West 1.39% (99% CI 1.11–1.75), Southeast 2.47% (99% CI 2.22–2.74), and South 2.01% (99% CI 1.66–2.43). State-level estimates, with their 99% CIs, are detailed in Table 1 and Figure 1A.

**Table 1:** Prevalence of anti-HCV positivity and viremia (confirmed and adjusted), RNA testing coverage and positivity, by regions and states.

Location	N	Anti-HCV+, n	Anti-HCV+ prevalence, % (99% CI)	HCV-RNA testing coverage, n/N (%)	HCV-RNA+ among those tested, % (n/N)	Confirmed HCV-RNA prevalence, % (99% CI)	Adjusted HCV-RNA prevalence (MAR), % (99% CI)
Brazil	66,601	1,385	2.1 (1.94–2.23)	906/1,385 (65.4)	64.2 (582/906)	0.9 (0.79–0.97)	1.3 (1.21–1.46)
North	3,609	102	2.8 (2.20–3.63)	48/102 (47.1)	43.8 (21/48)	0.6 (0.33–1.01)	1.2 (0.63–1.84)
Northeast	20,768	379	1.8 (1.60–2.08)	239/379 (63.1)	56.5 (135/239)	0.7 (0.52–0.81)	1.0 (0.83–1.23)
Central-West	8,983	125	1.4 (1.11–1.75)	72/125 (57.6)	65.3 (47/72)	0.5 (0.36–0.76)	0.9 (0.62–1.20)
Southeast	24,367	601	2.5 (2.22–2.74)	424/601 (70.5)	72.2 (306/424)	1.3 (1.08–1.45)	1.8 (1.55–2.01)
South	8,874	178	2.0 (1.66–2.43)	123/178 (69.1)	59.3 (73/123)	0.8 (0.61–1.11)	1.2 (0.87–1.51)
AC	452	38	8.4 (5.63–12.40)	3/38 (7.9)	66.7 (2/3)	0.4 (0.09–2.23)	5.6 (0.09–11.01)
AM	1,371	30	2.2 (1.38–3.46)	26/30 (86.7)	38.5 (10/26)	0.7 (0.33–1.60)	0.8 (0.18–1.51)
AP	361	6	1.7 (0.61–4.46)	4/6 (66.7)	50 (2/4)	0.6 (0.11–2.78)	0.8 (0.00–2.21)
PA	982	23	2.3 (1.38–3.94)	13/23 (56.5)	38.5 (5/13)	0.5 (0.17–1.51)	0.9 (0.00–1.84)
TO	90	1	1.1 (0.13–8.81)	1/1 (100)	100 (1/1)	1.1 (0.13–8.81)	1.1 (0.00–3.96)
RO	353	4	1.1 (0.34–3.73)	1/4 (25)	100 (1/1)	0.3 (0.03–2.37)	1.1 (0.00–2.58)
AL	1,991	43	2.2 (1.47–3.17)	38/43 (88.4)	13.2 (5/38)	0.3 (0.08–0.75)	0.3 (0.00–0.61)
BA	5,996	103	1.7 (1.34–2.21)	44/103 (42.7)	72.7 (32/44)	0.5 (0.34–0.84)	1.2 (0.82–1.68)
CE	3,177	53	1.7 (1.18–2.36)	36/53 (67.9)	52.8 (19/36)	0.6 (0.33–1.07)	0.9 (0.41–1.35)
MA	2,510	64	2.5 (1.86–3.49)	46/64 (71.9)	71.7 (33/46)	1.3 (0.84–2.04)	1.8 (1.10–2.56)
PB	1,147	37	3.2 (2.13–4.86)	27/37 (73)	51.9 (14/27)	1.2 (0.62–2.38)	1.7 (0.61–2.73)
PE	3,234	64	2.0 (1.44–2.72)	43/64 (67.2)	67.4 (29/43)	0.9 (0.56–1.44)	1.3 (0.77–1.89)
PI	549	6	1.1 (0.40–2.95)	0/6 (0)	—	—	—
RN	1,239	6	0.5 (0.18–1.32)	3/6 (50)	100 (3/3)	0.2 (0.06–0.95)	0.5 (0.00–0.99)
SE	925	3	0.3 (0.08–1.27)	2/3 (66.7)	0 (0/2)	—	—
DF	2,765	23	0.8 (0.49–1.41)	12/23 (52.2)	58.3 (7/12)	0.3 (0.10–0.65)	0.5 (0.08–0.89)
GO	3,118	52	1.7 (1.17–2.37)	39/52 (75)	59 (23/39)	0.7 (0.43–1.25)	1.0 (0.50–1.47)
MS	1,344	14	1.0 (0.53–2.03)	2/14 (14.3)	100 (2/2)	0.1 (0.03–0.76)	1.0 (0.33–1.76)
MT	1,756	36	2.1 (1.34–3.12)	19/36 (52.8)	78.9 (15/19)	0.9 (0.45–1.63)	1.6 (0.77–2.47)
ES	1,997	33	1.7 (1.06–2.56)	10/33 (30.3)	60 (6/10)	0.3 (0.11–0.82)	1.0 (0.20–1.78)
MG	7,750	114	1.5 (1.16–1.87)	77/114 (67.5)	42.9 (33/77)	0.4 (0.27–0.66)	0.6 (0.37–0.89)
RJ	3,816	126	3.3 (2.63–4.13)	88/126 (69.8)	68.2 (60/88)	1.6 (1.13–2.18)	2.3 (1.59–2.91)
SP	10,804	328	3.0 (2.64–3.49)	249/328 (75.9)	83.1 (207/249)	1.9 (1.60–2.29)	2.5 (2.12–2.92)
PR	3,455	44	1.3 (0.87–1.87)	22/44 (50)	72.7 (16/22)	0.5 (0.25–0.87)	0.9 (0.45–1.40)
RS	2,014	57	2.8 (2.02–3.95)	51/57 (89.5)	45.1 (23/51)	1.1 (0.67–1.93)	1.3 (0.61–1.94)
SC	3,405	77	2.3 (1.69–3.02)	50/77 (64.9)	68 (34/50)	1.0 (0.65–1.54)	1.5 (0.95–2.13)

Abbreviations – AC: Acre; AM: Amazonas; AP: Amapá; PA: Pará; RO: Rondônia; TO: Tocantins; AL: Alagoas; BA: Bahia; CE: Ceará; MA: Maranhão; PB: Paraíba; PE: Pernambuco; PI: Piauí; RN: Rio Grande do Norte; SE: Sergipe; DF: Distrito Federal; GO: Goiás; MS: Mato Grosso do Sul; MT: Mato Grosso; ES: Espírito Santo; MG: Minas Gerais; RJ: Rio de Janeiro; SP: São Paulo; PR: Paraná; RS: Rio Grande do Sul; SC: Santa Catarina.

Among anti-HCV–positive individuals, 66% were male, with a median age of 61 years (IQR 52–68). The predominant etiology of chronic kidney disease was systemic arterial hypertension (40.8%), followed by diabetes mellitus (21.8%) and chronic glomerulonephritis (13.4%). Coinfection was reported in 2.4% (34/1,385) of the population; 1.9% (27/1,385) were coinfected with HIV and 0.5% (7/1,385) with HBV. Data for the anti-HCV–positive population, by region and state (UF), are available in Table 2.

**Table 2:** Clinical characteristics of anti-HCV–positive patients who underwent HCV-RNA testing, comparing HCV-RNA–positive versus HCV-RNA–negative individuals.

Characteristic	HCV-RNA+	HCV-RNA−	p
**n (group)**	582	324	—
**Age, median [IQR]**	60 [52–68]	61 [52–69]	0.7903
Sex			0.5501
Female	191 (32.8%)	115 (35.5%)	
Male	390 (67.0%)	209 (64.5%)	
Not reported	1 (0.2%)	0 (0.0%)	
**CKD etiology**			0.2687
Hypertension	245 (42.1%)	123 (38.0%)	
Diabetes mellitus	112 (19.2%)	79 (24.4%)	
Chronic glomerulonephritis	92 (15.8%)	45 (13.9%)	
ADPKD	14 (2.4%)	13 (4.0%)	
Other	103 (17.7%)	57 (17.6%)	
Not reported	16 (2.7%)	7 (2.2%)	
**Dialysis modality**			0.5295
Hemodialysis	571 (98.1%)	321 (99.1%)	
Peritoneal dialysis	11 (1.9%)	3 (0.9%)	
**Presence of coinfection**			0.9982
Present	14 (2.4%)	8 (2.5%)	
Absent	568 (97.6%)	316 (97.5%)	
**ALT (U/L), median [IQR]**	14 [10–21]	13 [10–20]	0.0937

Abbreviations – ADPKD: autosomal dominant polycystic kidney disease; ALT: alanine aminotransferase; CKD: chronic kidney disease; HCV-RNA+: detectable HCV RNA (viremia present); HCV-RNA−: undetectable HCV RNA; IQR: interquartile range; U/L: units per liter; p: p-value (Mann–Whitney test for continuous variables; Pearson’s chi-square or Fisher’s exact test for categorical variables). Percentages are computed within each column (HCV-RNA+ or HCV-RNA−).

Among the 1,385 anti-HCV–positive individuals, 906 underwent HCV-RNA testing, corresponding to a coverage of 65.4%. Considering only anti-HCV–positive individuals with known results, HCV-RNA positivity was 64.2% (582/906). HCV-RNA coverage varied substantially across regions, with rates of 47.1% (48/102) in the North, 63.1% (239/379) in the Northeast, 57.6% (72/125) in the Central-West, 70.5% (424/601) in the Southeast, and 69.1% (123/178) in the South. State-level data, with their 99% CIs, are detailed in Table 1 and Figure 1B. The difference in coverage across regions was statistically significant (χ^2^ = 27.6; df = 4; *p* <0.001) (Figure S2). The 479 anti-HCV–positive individuals without an HCV-RNA result did not differ from the 906 tested in age (median 61 vs. 60 years; *p* = 0.70), sex (*p* = 0.91), or dialysis modality (99.0% vs. 98.5% on hemodialysis; *p* = 0.60), with a difference only in the distribution of chronic kidney disease etiology (*p* = 0.001).

HCV-RNA positivity was observed in 582 of 66,601 participants, corresponding to an (observed) prevalence of 0.87% (99% CI 0.79–0.97) (Figure 1C). Because not all anti-HCV–positive individuals underwent HCV-RNA testing, an adjusted prevalence was also calculated. Using this approach, national viremia was 1.34% (99% CI 1.21–1.46) (Figure 1D). Sensitivity analysis using extreme-case scenarios bounded the plausible range between 0.87% (assuming all untested anti-HCV–positive patients were HCV-RNA negative) and 1.59% (assuming all were HCV-RNA positive). Regionally, confirmed viremia estimates were 0.58% in the North (99% CI 0.33–1.01), 0.65% in the Northeast (99% CI 0.52–0.81), 0.52% in the Central-West (99% CI 0.36–0.76), 1.26% in the Southeast (99% CI 1.08–1.45), and 0.82% in the South (99% CI 0.61–1.11). After the MAR adjustment, estimates were 1.24% in the North (99% CI 0.63–1.84), 1.03% in the Northeast (99% CI 0.83–1.23), 0.91% in the Central-West (99% CI 0.62–1.20), 1.78% in the Southeast (99% CI 1.55–2.01), and 1.19% in the South (99% CI 0.87–1.51). State-level data, with their 99% CIs, are detailed in Table 1. Among HCV-RNA–positive patients, genotype information was available for 16.3% (95/582). Most had normal or mildly elevated ALT levels, with a trend toward a higher median in the HCV-RNA–positive group (14 vs. 13 U/L), although this difference was not statistically significant (*p* = 0.09) (Table 2).

**Figure 2:**
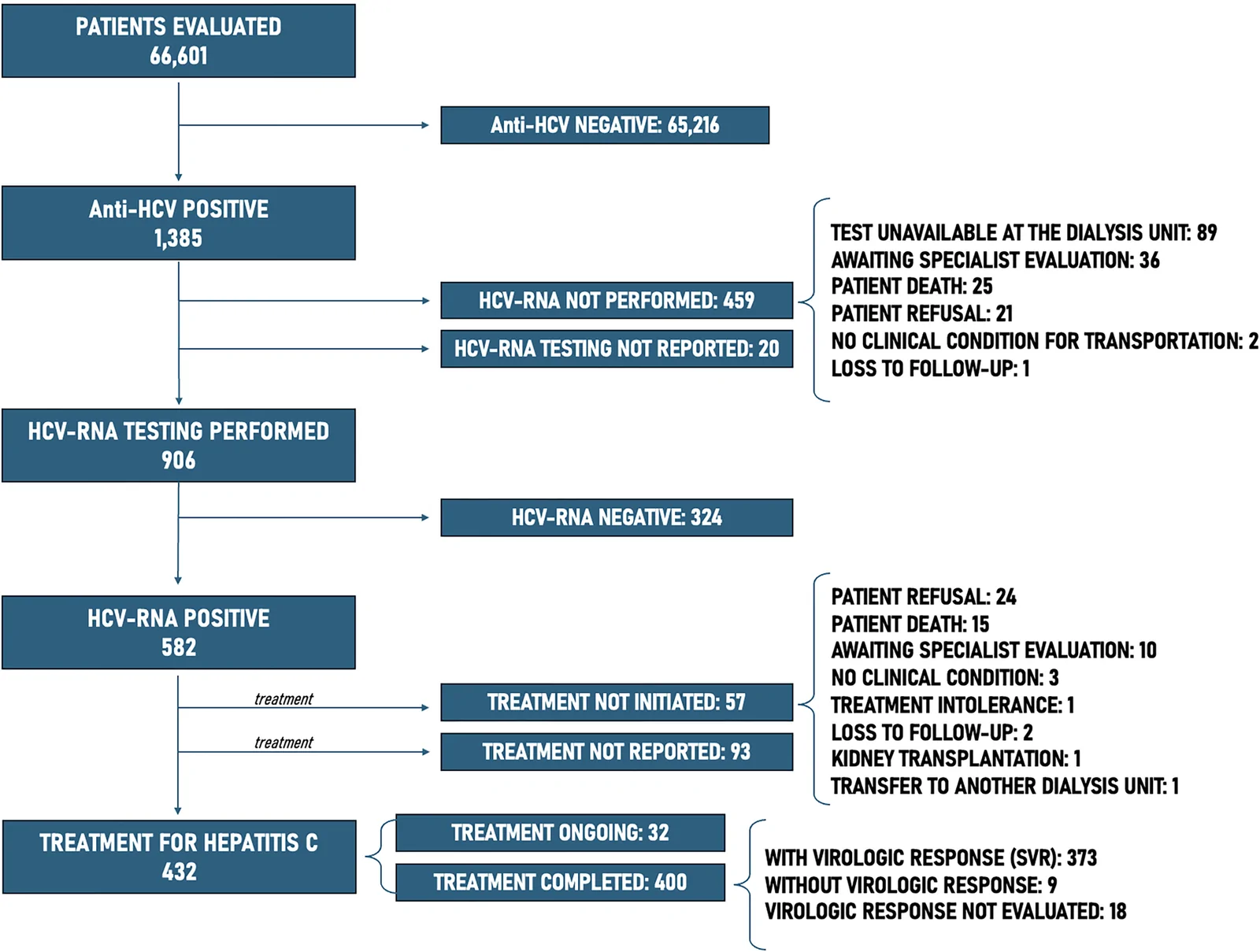
Cascade of care of the Brazilian Registry for the Elimination of Hepatitis C in Dialysis Units.

The national cascade of care (Figure 2) showed that, among the 582 patients with detectable viremia, 432 initiated treatment, corresponding to 74.2% of HCV-RNA–positive individuals. Among those treated, 400 completed the regimen, representing a treatment completion rate of 92.6%. Among those who completed treatment, sustained virologic response was documented in 373 individuals, equivalent to 93.3%. Data by region and state (UF) are detailed in Table 3.

**Table 3:** Cascade of care among HCV-RNA–positive patients: Brazil, regions, and federative units.

Stratum	Treated n*/N (%)	Treatment completed n/N (%)	SVR n/N (%)
BRAZIL	432/582 (74.2%)	400/432 (92.6%)	373/400 (93.3%)
NORTH	11/21 (52.4%)	10/11 (90.9%)	8/10 (80.0%)
NORTHEAST	93/135 (68.9%)	84/93 (90.3%)	75/84 (89.3%)
CENTRAL-WEST	18/47 (38.3%)	16/18 (88.9%)	14/16 (87.5%)
SOUTHEAST	259/306 (84.6%)	240/259 (92.7%)	233/240 (97.1%)
SOUTH	51/73 (69.9%)	50/51 (98.0%)	43/50 (86.0%)
AC	2/2 (100.0%)	2/2 (100.0%)	2/2 (100.0%)
AM	3/10 (30.0%)	3/3 (100.0%)	3/3 (100.0%)
AP	2/2 (100.0%)	2/2 (100.0%)	0/2 (0.0%)
PA	2/5 (40.0%)	2/2 (100.0%)	2/2 (100.0%)
RO	1/1 (100.0%)	1/1 (100.0%)	1/1 (100.0%)
TO	1/1 (100.0%)	0/1 (0.0%)	—
AL	5/5 (100.0%)	5/5 (100.0%)	5/5 (100.0%)
BA	23/32 (71.9%)	18/23 (78.3%)	17/18 (94.4%)
CE	13/19 (68.4%)	11/13 (84.6%)	10/11 (90.9%)
MA	23/33 (69.7%)	23/23 (100.0%)	19/23 (82.6%)
PB	12/14 (85.7%)	12/12 (100.0%)	12/12 (100.0%)
PE	14/29 (48.3%)	12/14 (85.7%)	9/12 (75.0%)
PI	—	—	—
RN	3/3 (100.0%)	3/3 (100.0%)	3/3 (100.0%)
SE	—	—	—
DF	6/7 (85.7%)	6/6 (100.0%)	5/6 (83.3%)
GO	9/23 (39.1%)	7/9 (77.8%)	6/7 (85.7%)
MS	1/2 (50.0%)	1/1 (100.0%)	1/1 (100.0%)
MT	2/15 (13.3%)	2/2 (100.0%)	2/2 (100.0%)
ES	6/6 (100.0%)	6/6 (100.0%)	6/6 (100.0%)
MG	16/33 (48.5%)	16/16 (100.0%)	16/16 (100.0%)
RJ	50/60 (83.3%)	42/50 (84.0%)	35/42 (83.3%)
SP	187/207 (90.3%)	176/187 (94.1%)	176/176 (100.0%)
PR	14/16 (87.5%)	14/14 (100.0%)	14/14 (100.0%)
RS	19/23 (82.6%)	19/19 (100.0%)	12/19 (63.2%)
SC	18/34 (52.9%)	17/18 (94.4%)	17/17 (100.0%)

Abbreviations – AC: Acre; AM: Amazonas; AP: Amapá; PA: Pará; RO: Rondônia; TO: Tocantins; AL: Alagoas; BA: Bahia; CE: Ceará; MA: Maranhão; PB: Paraíba; PE: Pernambuco; PI: Piauí; RN: Rio Grande do Norte; SE: Sergipe; DF: Distrito Federal; GO: Goiás; MS: Mato Grosso do Sul; MT: Mato Grosso; ES: Espírito Santo; MG: Minas Gerais; RJ: Rio de Janeiro; SP: São Paulo; PR: Paraná; RS: Rio Grande do Sul; SC: Santa Catarina.

Note – *N: HCV-RNA+; SVR: sustained virologic response.

Analysis of the reasons for attrition elucidated distinct bottlenecks at the stages of diagnostic confirmation and treatment initiation. Among the 1,385 anti-HCV–positive individuals, 459 did not undergo HCV-RNA testing. The recorded reasons were test unavailability in 89 cases (19.4%), awaiting specialist evaluation in 36 (7.8%), death in 25 (5.4%), refusal of testing in 21 (4.6%), inability to travel for sample collection due to clinical condition in 2 (0.4%), and loss to follow-up due to transfer to another dialysis unit in 1 (0.2%), in addition to 285 records reported as not informed (62.1%) (Figure 2). In addition, whether HCV-RNA testing was performed was not reported for 20 individuals.

Among the 582 HCV-RNA–positive patients, 57 did not initiate treatment. The most frequent causes were patient refusal of treatment (24; 42.1%), death (15; 26.3%), and awaiting evaluation by a specialist (10; 17.5%), followed by clinical inability to undergo treatment (3; 5.3%), lack of access to medication (2; 3.5%), intolerance (1; 1.8%), and loss to follow-up due to kidney transplantation (1; 1.8%) or transfer to another dialysis unit (1; 1.8%) (Figure 2). Treatment information was not reported for 93 individuals.

## DISCUSSION

Hepatitis C remains one of the leading causes of liver transplantation in several countries and is associated with substantial costs related to hospitalizations and the treatment of chronic complications. Individuals infected with HCV have a two- to three-fold higher risk of hospitalization and mortality from hepatic and extrahepatic causes, including cardiovascular, renal, and lymphoproliferative diseases. Globally, regions such as Eastern Europe and Central Asia still have a higher prevalence, whereas countries in North America and Western Europe have observed significant reductions following the introduction of DAAs and aggressive screening and early treatment policies, particularly in higher-risk populations such as patients on dialysis^1,2^.

In Brazil, the Brazilian Dialysis Census shows a marked decline in anti-HCV antibody prevalence, from 19.3% in 2000 to 2.1% in 2023. However, the diagnosis of active infection—dependent on detection of viremia by HCV-RNA—remains underused in clinical practice. The absence of this laboratory confirmation favors the progression of liver disease in infected individuals, increases the risk of seroconversion among patients, and contributes to occupational exposure among healthcare workers^4,7^. Data from the Centers for Disease Control and Prevention (CDC) indicate that more than half of hepatitis C outbreaks between 2008 and 2015 occurred in hemodialysis settings, associated with multiple factors: poor adherence to or breaches of standard precautions, lack of early detection of suspected cases, and delays in referral for treatment. In addition, there are no vaccines available against HCV, and no post-exposure prophylaxis exists. In Brazil, the containment of an outbreak in a chronic kidney care hospital unit in the Triângulo Mineiro illustrates the feasibility of local micro-elimination: active case finding, tracing of transmission routes, correction of routine techniques, and treatment of all reactive cases interrupted transmission^11^.

Currently, eliminating hepatitis C in dialysis units is an attainable goal and requires universal, periodic screening, laboratory confirmation of active infection, timely initiation of treatment, and strict adherence to biosafety measures^4,12^. Recent systematic reviews indicate a global prevalence in the general population ranging from 1% to 1.8%, depending on method, region, sample size, and period analyzed (pre- or post-DAA era)^13^. In dialysis patients, anti-HCV antibody prevalence is substantially higher than in the general population, reflecting the increased transmission risk associated with hemodialysis. Recent meta-analyses have shown an average prevalence between 5% and 10%, with wide regional and temporal variability. Countries with rigorous infection control protocols and expanded access to antiviral therapy have reported a marked decline in these rates^14,15,16^.

This multicenter registry represents the first coordinated nationwide effort to characterize the prevalence, the cascade of care, and the barriers to eliminating HCV among dialysis patients in Brazil. The inclusion of more than 66,000 individuals from 384 dialysis units provides statistical robustness to the estimates and nationwide breadth to the portrait of the care pathway, reflecting the regional and structural heterogeneity of the Brazilian dialysis network.

All individuals were tested for anti-HCV, with a positivity rate of 2.1% (1,385 people). HCV-RNA coverage among these individuals was 65.4% and ranged from 47% in the North to 70% in the Southeast. The main reasons for missing test results in the spreadsheets were the unavailability of sample collection at the dialysis unit itself, difficulty or unavailability of referral to a specialist, refusal to undergo testing, or simply lack of information in the medical record. These findings reflect operational bottlenecks in confirming active infection but also signal concrete opportunities for process interventions, such as incorporating reflex-testing routines (anti-HCV positivity with automatic reflex to HCV-RNA), with sample collection at the dialysis unit; simplified regulatory pathways for specialist evaluation; provision of treatment within the dialysis unit; and measures to strengthen engagement of the healthcare team.

Among anti-HCV–positive individuals, the proportion of patients with detectable viremia (HCV-RNA positive) varies widely. In the general population, approximately 70% of individuals will develop chronic infection, with persistently positive HCV-RNA^3^. Among dialysis patients, this proportion is highly variable, ranging from 30% to 65% depending on the center studied^17^. In this study, HCV-RNA positivity was 64.2% (582/906). From a programmatic perspective, assuming that untested individuals have the same positivity rate as those tested, approximately 6 in 10 dialysis patients who were anti-HCV positive had active infection (HCV-RNA positive). Therefore, HCV-RNA testing is essential to confirm active infection, as recommended by the main national and international guidelines^4,5,9^. The national prevalence of viremia among dialysis patients, around 1.34%, places Brazil at a level similar to that of countries that have advanced substantially in HCV micro-elimination in dialysis. In Taiwan, Huang et al.^6^ demonstrated a reduction in viremia from 7.7% to 0.6% in only three years of active surveillance, whereas Hu et al.^18^ achieved 98% diagnostic coverage after implementing a collaborative model involving nephrologists, hepatologists, and public health managers.

The cascade of care was clinically robust: 74.2% of patients with detectable viremia initiated treatment (432/582), 92.6% completed therapy (400/432), and 93.3% achieved SVR (373/400). Direct-acting antivirals have demonstrated high efficacy and safety for treating hepatitis C in dialysis patients, achieving sustained virologic response rates (SVR12 93–98%) in most series^4,5,19^. Attrition before initiation of DAA therapy was mainly explained by refusal (42.1%), death (26.3%), and awaiting specialist evaluation (17.5%). This suggests two axes for public health response: (i) adherence and education interventions (to reduce refusals and support shared decision-making) and (ii) timely access to evaluation/therapy (to minimize delays and fatal outcomes before initiation). Death as a reason for non-treatment reinforces the need to shorten the time between diagnosis and DAA initiation in dialysis settings. There were no statistically significant differences in sex, age, dialysis modality, or ALT between the groups with and without viremia. Genotype data were available for only 16.3% of HCV-RNA–positive patients, which limits specific inferences.

Our findings indicate that HCV elimination in dialysis populations is feasible when care integration and institutional support are in place. In the Brazilian context, treatment initiation rates (74%) and sustained virologic response (SVR, 93%) among those who completed treatment reinforce the effectiveness and safety of DAAs. HCV micro-elimination in dialysis units is achievable. To this end, periodic universal screening (anti-HCV testing with confirmation by HCV-RNA), an agile pathway for treatment initiation, and monitoring of the following indicators are necessary: % tested, % HCV-RNA–positive patients referred, % initiating DAA, SVR^14^, new incident cases within the unit, and integration of the multidisciplinary team for treatment logistics and monitoring. The experience from Taiwan^6,18^ indicates that direct coordination of the public health system, combined with the training of multidisciplinary teams and the standardization of screening and treatment, enabled achievement of the micro-elimination indicators recommended by the WHO^3^.

The success of the indicators observed in this national registry is inseparable from the public health model adopted in Brazil. Since the 1988 Constitution, health has been established as a fundamental right of all and a duty of the State, which allowed for the structuring of the Unified Health System (SUS) under the principles of universality and equity. In the context of hepatitis C, this guideline translates into a pioneering public health policy, in which the Ministry of Health ensures the free provision of direct-acting antivirals for the entire dialysis population undergoing renal replacement therapy^9^. This universal and uninterrupted access to high-cost medications eliminates one of the most critical financial barriers to viral eradication, making HCV micro-elimination a feasible goal and positioning Brazil as an example of institutional commitment to the health of vulnerable groups.

In Brazil, the model led by the SBN in partnership with the SBH and IBRAFIG follows the same principle, promoting synergy among specialists and public managers to achieve elimination targets in dialysis units. The findings suggest that Brazil is at an advanced stage toward eliminating HCV in the dialysis population. Considering an adjusted viremia prevalence of 1.34% and the current estimated dialysis population (approximately 160,000 people), the number of individuals with active infection would be fewer than 2,000, a fully treatable population within the operational capacity of the SUS. The expected impact of viral elimination includes improved quality of life, reduced hospitalizations and cardiovascular and liver-related mortality, and elimination of the need to isolate dialysis machines for infected patients. The concept of “treatment as prevention,” reinforced by the FORMOSA-LIKE study^6^, underscores the importance of universal treatment as a collective prevention tool. Eradicating the virus within dialysis units reduces not only the risk of reinfection and new cases but also the burden of stigma associated with infection.

The regional distribution of participants reflects the voluntary participation of the units, not population demographics or the national distribution of dialysis patients. Relative to the national network, the Southeast—which concentrates about 47% of the country’s active units—was under-represented (33.3% of centers and 36.6% of the Registry’s patients), whereas the Northeast and Central-West were over-represented, owing to greater mobilization in those regions (Figure S1). This heterogeneity of participation explains, for example, why the number of participants in the Northeast (20,768) exceeds that in the South (8,874).

This study has limitations. First, although patient enrollment within each unit was census-based, unit participation was voluntary (384 of 904 active units; 42.5%), so the set of centers constitutes a non-probability sample of services, possibly subject to self-selection bias, whereby units with better infrastructure or greater team engagement tend to join such initiatives preferentially. Prevalence estimates should therefore be read as descriptive of the enrolled set, not as weighted national parameters, and caution is warranted in direct extrapolation, especially to under-represented regions. As noted by Niquini *et al.* ^8^, prevalence estimates of HCV in dialysis are particularly sensitive to selection and publication biases. Second, HCV-RNA verification was incomplete (65.4% coverage) and differential across regions. We mitigated this limitation through the MAR-adjusted estimate, the sensitivity analysis that bounded viremia between 0.87% and 1.59% (Figure S3), and the demonstration that untested patients were similar to tested patients in the main observable characteristics (age, sex, and modality), which makes relevant bias due to non-random missingness unlikely, although it does not exclude it. We acknowledge, however, that the MAR assumption is not directly testable. Third, genotype information was available for only 16.3% of viremic cases, limiting inferences about the genotypic distribution. Finally, long-term clinical outcomes and the incidence of new cases per unit were not collected, which should be addressed in the transformation of the Registry into a permanent surveillance system. It should be noted that the therapeutic-effectiveness indicators (initiation, completion, and SVR) refer to patients who were actually treated and are therefore robust to the question of center representativeness.

This study represents the first national initiative for micro-elimination of hepatitis C among patients receiving dialysis therapy, spanning an entire country of continental dimensions and marked by substantial regional heterogeneity in access, diagnostic infrastructure, and care organization. The Brazilian experience demonstrates that eliminating hepatitis C in dialysis units is a feasible goal when supported by three pillars: universal, periodic screening; immediate treatment of confirmed cases; and strict adherence to biosafety practices. Cooperation among medical societies and public health managers, already consolidated through the Brazilian Registry for the Elimination of Hepatitis C in Dialysis Units, constitutes an effective model for integrating nephrology and hepatology in addressing a persistent public health problem. Transforming this registry into a permanent surveillance and monitoring system may consolidate HCV micro-elimination and strengthen the quality of care in dialysis units. Brazil has the therapeutic resources, the institutional structure, and the technical capacity required to eradicate HCV in the dialysis population. The remaining challenge is essentially operational: ensuring that every patient is tested, treated, and cured and that each dialysis unit becomes, in fact, an environment free of hepatitis C.

## Data Availability

The datasets generated and/or analyzed during the present study are not publicly available due to ethical and privacy restrictions, but may be obtained from the corresponding author upon reasonable request.

## SUPPLEMENTARY MATERIAL

The following online material is available for this article:

Figure S1 – Regional representativeness of the Registry relative to the national dialysis network. (A) Percentage distribution of participating centers in the Registry versus active dialysis units in Brazil (SBN Brazilian Dialysis Census). (B) Percentage distribution of patients included in the Registry versus the national distribution. The differences reflect the voluntary participation of the units, not population demographics.

Figure S2 – HCV-RNA testing coverage among anti-HCV–positive patients, by region, with 95% confidence intervals (Wilson method). The dashed line indicates national coverage (65.4%). Heterogeneity across regions was statistically significant (χ^2^ = 27.6; df = 4; *p* < 0.001).

Figure S3 – Sensitivity analysis of active viremia (HCV-RNA+) prevalence in the dialysis population. The confirmed estimate (0.87%) corresponds to the lower bound (all untested assumed negative) and the maximum scenario (1.59%), to the upper bound (all positive). The adjusted (MAR) estimate of 1.34% lies within the plausible range delimited by these extremes. Bars indicate 99% CIs.
