# Supplementary material for: Microelimination of hepatitis C in dialysis units: results from the Brazilian Registry for the elimination of HCV
Source: J Bras Nefrol. 2026 Sep 14;48(4):e20260092. doi: 10.1590/2175-8239-JBN-2026-0092en (PMC13577599; doi:10.1590/2175-8239-JBN-2026-0092en)
Supplement: Supplementary file 1 [file 2175-8239-jbn-48-4-e20260092-suppl1.pdf]

Braz. J. Nephrol. 2026
DOI: https://doi.org/10.1590/2175-8239-JBN-2026-0092en

Supplementary Material to "Microelimination of hepatitis C in dialysis units: results from the Brazilian Registry for HCV elimination"

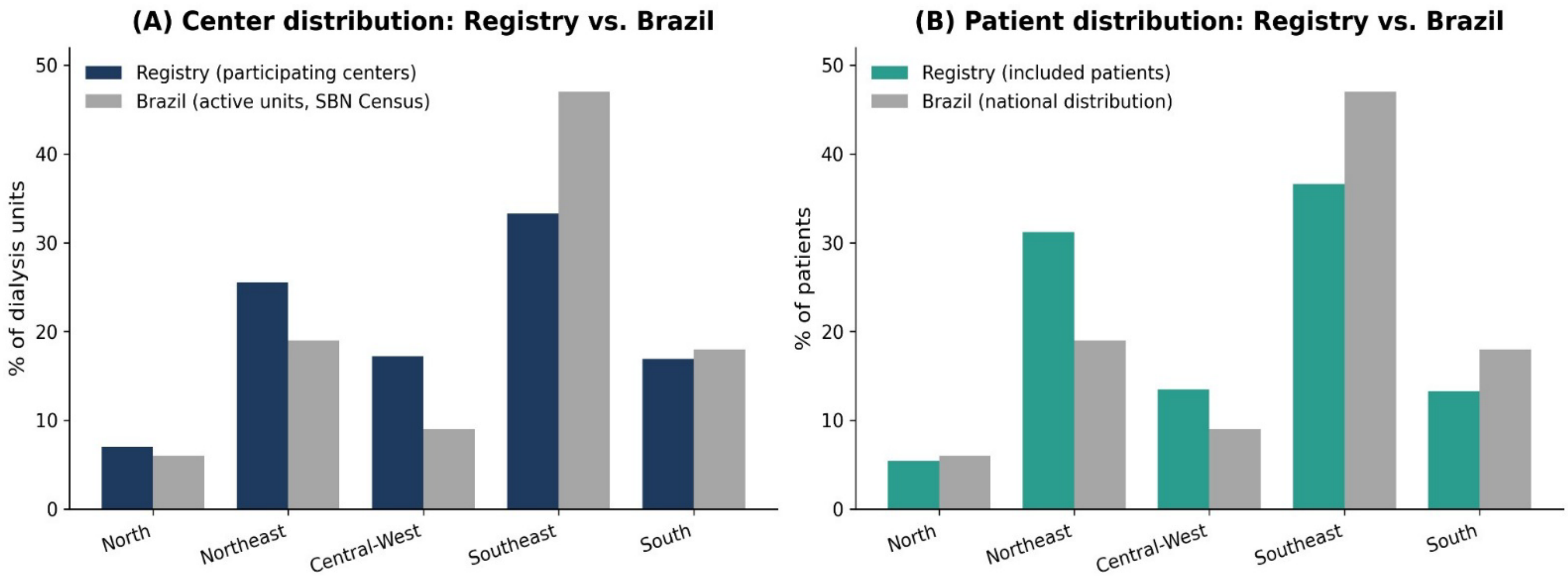

Figure S1. Regional representativeness of the Registry relative to the national dialysis network. (A) Percentage distribution of participating centers in the Registry versus active dialysis units in Brazil (SBN Brazilian Dialysis Census). (B) Percentage distribution of patients included in the Registry versus the national distribution. The differences reflect the voluntary participation of the units, not population demographics.
