# Supplementary material for: Microelimination of hepatitis C in dialysis units: results from the Brazilian Registry for the elimination of HCV
Source: J Bras Nefrol. 2026 Sep 14;48(4):e20260092. doi: 10.1590/2175-8239-JBN-2026-0092en (PMC13577599; doi:10.1590/2175-8239-JBN-2026-0092en)
Supplement: Supplementary file 2 [file 2175-8239-jbn-48-4-e20260092-suppl2.pdf]

Braz. J. Nephrol. 2026
DOI: https://doi.org/10.1590/2175-8239-JBN-2026-0092en

Supplementary Material to "Microelimination of hepatitis C in dialysis units: results from the Brazilian Registry for HCV elimination"

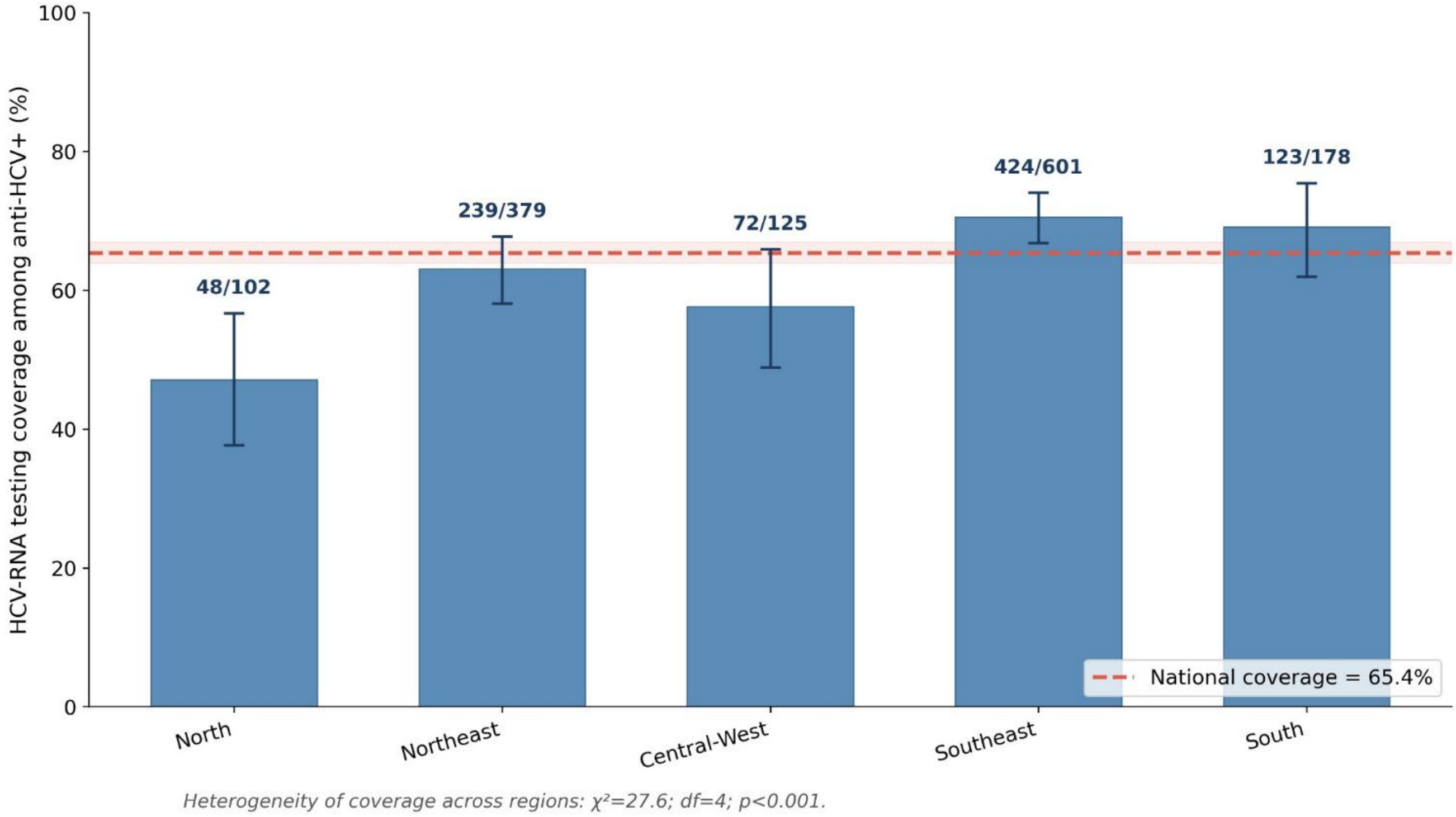

Figure S2. HCV-RNA testing coverage among anti-HCV–positive patients, by region, with 95% confidence intervals (Wilson method). The dashed line indicates national coverage (65.4%). Heterogeneity across regions was statistically significant (χ^2=27.6; df=4; p<0.001).
