# Supplementary material for: Microelimination of hepatitis C in dialysis units: results from the Brazilian Registry for the elimination of HCV
Source: J Bras Nefrol. 2026 Sep 14;48(4):e20260092. doi: 10.1590/2175-8239-JBN-2026-0092en (PMC13577599; doi:10.1590/2175-8239-JBN-2026-0092en)
Supplement: Supplementary file 3 [file 2175-8239-jbn-48-4-e20260092-suppl3.pdf]

Braz. J. Nephrol. 2026
DOI: https://doi.org/10.1590/2175-8239-JBN-2026-0092en

Supplementary Material to "Microelimination of hepatitis C in dialysis units: results from the Brazilian Registry for HCV elimination"

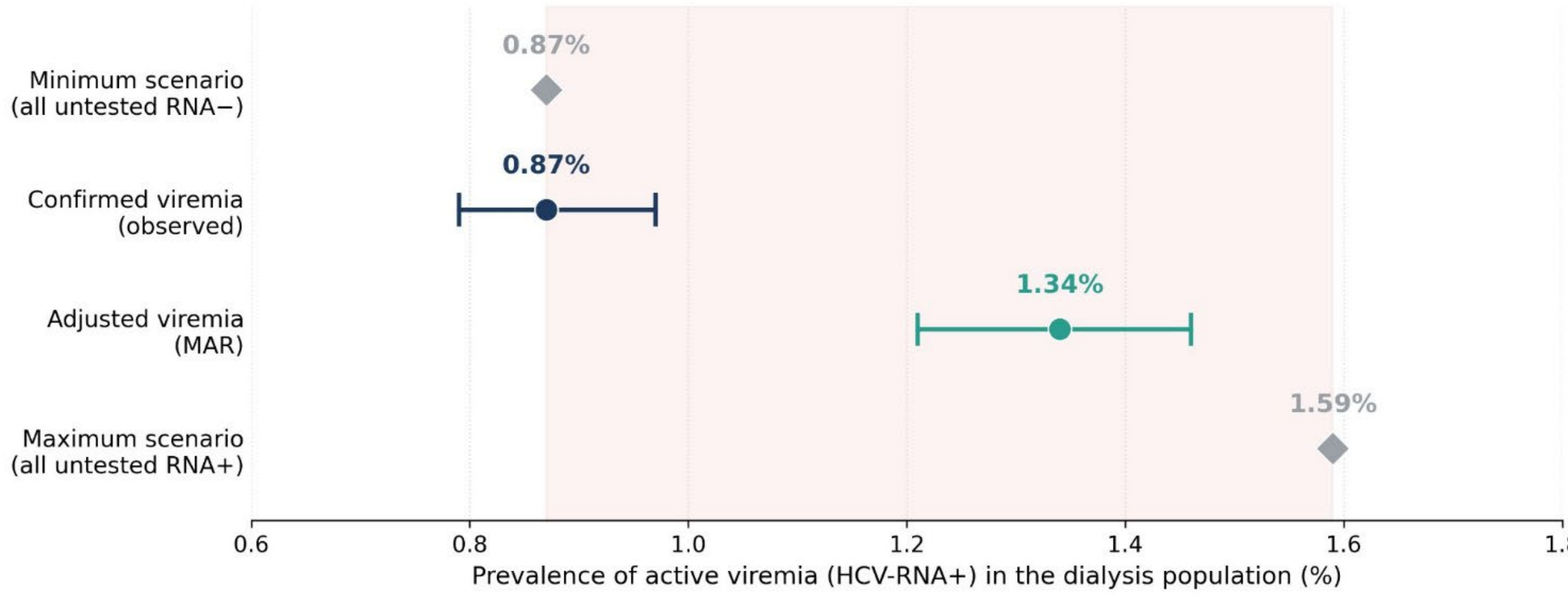

Figure S3. Sensitivity analysis of active viremia (HCV-RNA+) prevalence in the dialysis population. The confirmed estimate (0.87%) corresponds to the lower bound (all untested assumed negative) and the maximum scenario (1.59%) to the upper bound (all positive). The adjusted (MAR) estimate of 1.34% lies within the plausible range delimited by these extremes. Bars indicate 99% CIs.
