# Supplementary material for: Microelimination of hepatitis C in dialysis units: results from the Brazilian Registry for the elimination of HCV
Source: J Bras Nefrol. 2026 Sep 14;48(4):e20260092. doi: 10.1590/2175-8239-JBN-2026-0092en (PMC13577599; doi:10.1590/2175-8239-JBN-2026-0092en)
Supplement: Supplementary file 4 [file 2175-8239-jbn-48-4-e20260092-suppl4.pdf]

Braz. J. Nephrol. 2026
DOI: https://doi.org/10.1590/2175-8239-JBN-2026-0092pt

Material Suplementar para "Microeliminação da hepatite C em unidades de diálise: resultados do Registro Brasileiro para eliminação do HCV"

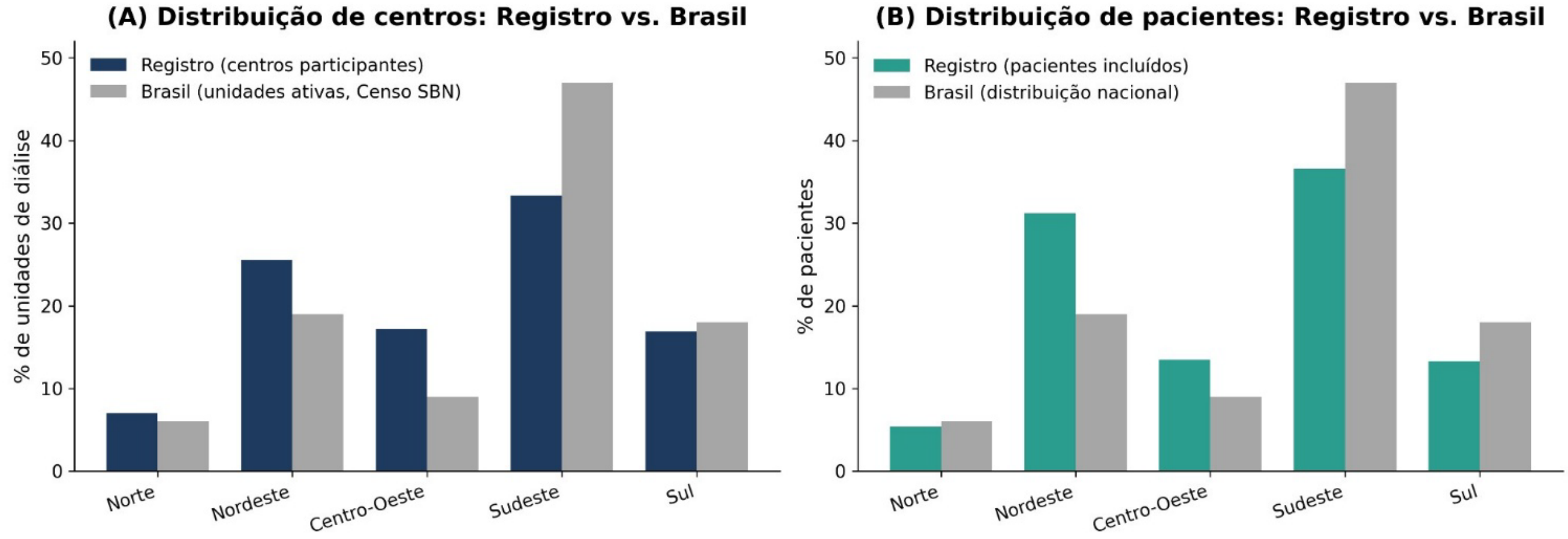

Figura S1. Representatividade regional do Registro frente às Unidades de diálise. (A) Distribuição percentual de centros participantes do Registro versus unidades de diálise ativas no Brasil (Censo Brasileiro de Diálise da SBN). (B) Distribuição percentual de pacientes incluídos no Registro versus distribuição nacional. As diferenças refletem a adesão voluntária das unidades, e não a demografia populacional.
