# Supplementary material for: Microelimination of hepatitis C in dialysis units: results from the Brazilian Registry for the elimination of HCV
Source: J Bras Nefrol. 2026 Sep 14;48(4):e20260092. doi: 10.1590/2175-8239-JBN-2026-0092en (PMC13577599; doi:10.1590/2175-8239-JBN-2026-0092en)
Supplement: Supplementary file 5 [file 2175-8239-jbn-48-4-e20260092-suppl5.pdf]

Braz. J. Nephrol. 2026
DOI: https://doi.org/10.1590/2175-8239-JBN-2026-0092pt

Material Suplementar para "Microeliminação da hepatite C em unidades de diálise: resultados do Registro Brasileiro para eliminação do HCV"

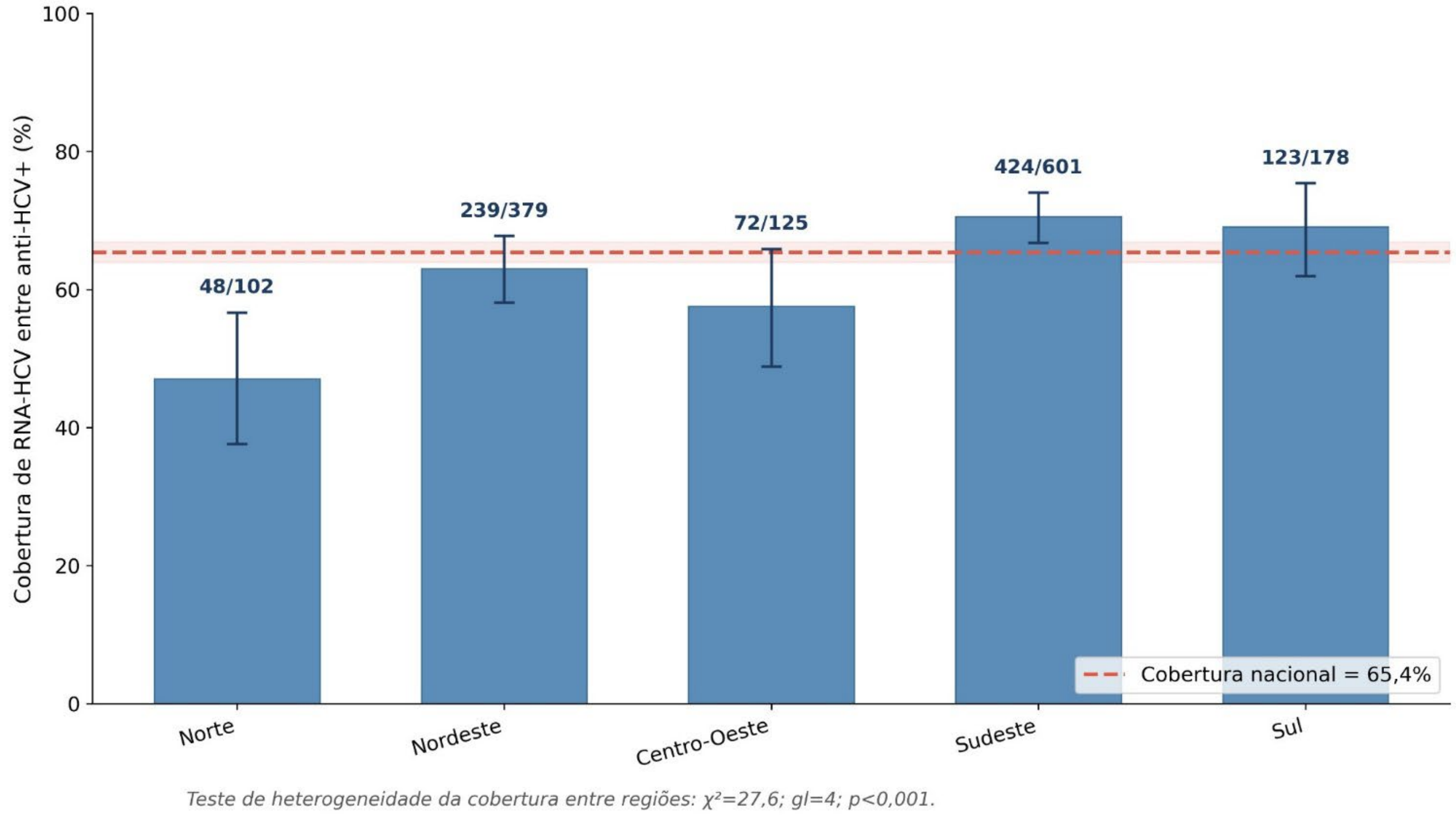

Figura S2. Cobertura do teste de RNA-HCV entre pacientes anti-HCV positivos, por região, com intervalos de confiança de 95% (método de Wilson). A linha tracejada indica a cobertura nacional (65,4%). A heterogeneidade entre regiões foi estatisticamente significativa (χ^2=27,6; gl=4; p<0,001).
