# Supplementary material for: Microelimination of hepatitis C in dialysis units: results from the Brazilian Registry for the elimination of HCV
Source: J Bras Nefrol. 2026 Sep 14;48(4):e20260092. doi: 10.1590/2175-8239-JBN-2026-0092en (PMC13577599; doi:10.1590/2175-8239-JBN-2026-0092en)
Supplement: Supplementary file 6 [file 2175-8239-jbn-48-4-e20260092-suppl6.pdf]

Braz. J. Nephrol. 2026

DOI: https://doi.org/10.1590/2175-8239-JBN-2026-0092pt

Material Suplementar para "Microeliminação da hepatite C em unidades de diálise: resultados do Registro Brasileiro para eliminação do HCV"

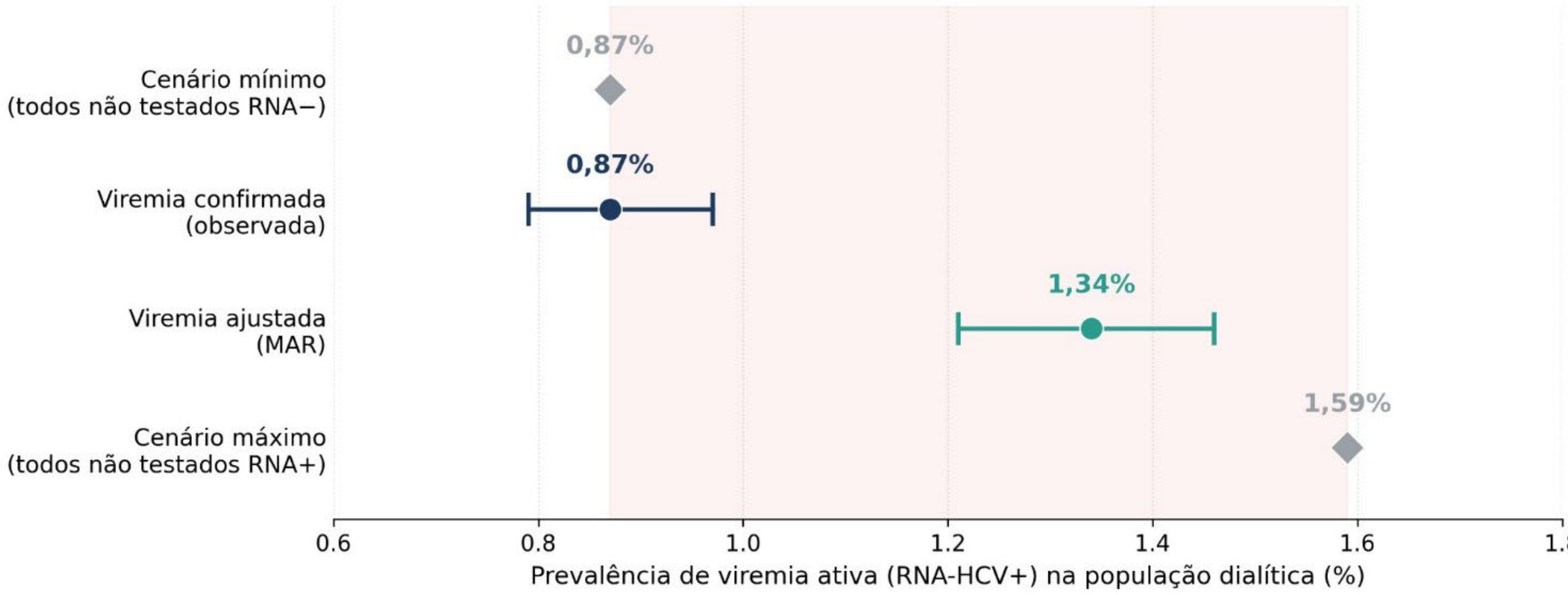

Figura S3. Análise de sensibilidade da prevalência de viremia ativa (RNA-HCV+) na população dialítica. A estimativa confirmada (0,87%) corresponde ao limite inferior (todos os não testados assumidos como negativos) e o cenário máximo (1,59%) ao limite superior (todos positivos). A estimativa ajustada (MAR) de 1,34% situa-se na faixa plausível delimitada por esses extremos. Barras indicam IC 99%.
