# Supplementary material for: Microelimination of hepatitis C in dialysis units: results from the Brazilian Registry for the elimination of HCV
Source: J Bras Nefrol. 2026 Sep 14;48(4):e20260092. doi: 10.1590/2175-8239-JBN-2026-0092en (PMC13577599; doi:10.1590/2175-8239-JBN-2026-0092en)
Supplement: Translation

# Translation: Microeliminação da hepatite C em unidades de diálise: resultados do Registro Brasileiro para eliminação do HCV

**DOI:** 10.1590/2175-8239-JBN-2026-0092pt

**Authors:** Patrícia Ferreira Abreu, Pablo Rodrigues Costa Alves, Paulo Lisboa Bittencourt, Isadora Cartaxo de Sousa Calvo, Osvaldo Merege Vieira, Marcelo Mazza do Nascimento, Felipe Costa Neves, Ana Flavia Moura, Daniel Costa Chalabi Calazans, Juan Miguel Villalobos Salcedo, Cristina Melo Rocha, Cirley Maria de Oliveira Lobato, Alex Vianey Callado França, Alessandra Maria Mont’Alverne Pierre, Adalgisa de Souza Paiva Ferreira, Edmundo Pessoa Lopes, Jose Eymard Moraes de Medeiros, Francisco José Dutra Souto, Rodrigo Sebba Aires, Liliana Sampaio Costa Mendes, Silvia Naomi de Oliveira Uehara, Luciana Lofego Gonçalves, Lilian Pires de Freitas do Carmo, Cláudia Alexandra Pontes Ivantes, Hugo Cheinquer, Tatiara Bueno Parreira, Karla Cristina Silva Petruccelli Israel, Verônica de Jesus Rodrigues Cardozo Costa, Hivis da Costa Sousa, Jarinne Camilo Landim Nasserala, Kleyton de Andrade Bastos, Maria Eliete Pinheiro, Artur Quintiliano Bezerra da Silva, Claudia Maria Costa de Oliveira, Flávio Henrique Soares Barros, Marclébio Manuel Coêlho Dourado, Ciro Bruno Costa, Ricardo Araújo Mothé, Alexandre Silvestre Cabral, Denise Rodrigues Simão, Fellype de Carvalho Barreto, Paulo Henrique Fraxino, Natasha Silva Constancio, Dirceu Reis da Silva, Álvaro Pacheco Silva, Lúcio Roberto Requião Moura, David Jose de Barros Machado, Alexandre Minetto Brabo, Alice Pignaton Naseri, Tomás Ribeiro Carvalho, Nathalia da Fonseca Pestana, Pedro Tulio Monteiro de Castro e Abreu Rocha, Leila Maria Soares Tojal de Barros Lima, Renata M. Perez, Janaina Luz Narciso-Schiavon, Antônio Ricardo Cardia Ferraz de Andrade, Bruno Zawadzki, José A. Moura-Neto, Maria Lucia Gomes Ferraz

**Affiliations:** 1 Sociedade Brasileira de Nefrologia, São Paulo, SP, Brasil.; 2 Universidade Federal de São Paulo, São Paulo, SP, Brasil.; 3 Universidade Federal da Paraíba, João Pessoa, PB, Brasil.; 4 Centro Municipal de Nefrologia de João Pessoa, João Pessoa, PB, Brasil.; 5 Sociedade Brasileira de Hepatologia, São Paulo, SP, Brasil.; 6 Instituto Brasileiro do Fígado, São Paulo, SP, Brasil.; 7 Escola Bahiana de Medicina e Saúde Pública, Salvador, BA, Brasil.; 8 Hospital Português, Salvador, BA, Brasil.; 9 Universidade de São Paulo, Faculdade de Medicina de Ribeirão Preto, Ribeirão Preto, SP, Brasil.; 10 Universidade Federal do Paraná, Curitiba, PR, Brasil.; 11 Universidade Federal de Rondônia, Porto Velho, RO, Brasil.; 12 Universidade do Estado do Amazonas, Manaus, AM, Brasil.; 13 Universidade Federal do Acre, Rio Branco, AC, Brasil.; 14 Secretaria Estadual de Saúde do Acre, Rio Branco, AC, Brasil.; 15 Universidade Federal de Sergipe, Aracaju, SE, Brasil.; 16 Hospital Geral de Fortaleza, Fortaleza, CE, Brasil.; 17 Universidade Federal do Maranhão, São Luís, MA, Brasil.; 18 Universidade Federal de Pernambuco, Hospital das Clínicas, Recife, PE, Brasil.; 19 Universidade Federal de Pernambuco, Recife, PE, Brasil.; 20 Universidade Federal de Mato Grosso, Cuiabá, MT, Brasil.; 21 Universidade Federal de Goiás, Goiânia, GO, Brasil.; 22 Hospital de Base do Distrito Federal, Brasília, DF, Brasil.; 23 Universidade Federal de Mato Grosso do Sul, Campo Grande, MS, Brasil.; 24 Universidade Federal do Espírito Santo, Vitória, ES, Brasil.; 25 Universidade Federal de Minas Gerais, Belo Horizonte, MG, Brasil.; 26 Hospital de Clínicas de Porto Alegre, Porto Alegre, RS, Brasil.; 27 Universidade Federal do Amazonas, Manaus, AM, Brasil.; 28 Universidade Federal do Amapá, Hospital Universitário, Macapá, AP, Brasil.; 29 Hospital do Rim do Acre, Rio Branco, AC, Brasil.; 30 Universidade Federal de Alagoas, Maceió, AL, Brasil.; 31 Universidade Federal do Rio Grande do Norte, Natal, RN, Brasil.; 32 Universidade Federal do Ceará, Hospital Universitário Walter Cantídio, Fortaleza, CE, Brasil.; 33 Hospital Santa Isabel, Blumenau, SC, Brasil.; 34 Conselho Regional de Medicina do Paraná, Câmara Técnica de Nefrologia, Curitiba, PR, Brasil.; 35 Associação Renal Vida, Blumenau, SC, Brasil.; 36 Universidade de São Paulo, Faculdade de Medicina, Hospital das Clínicas, São Paulo, SP, Brasil.; 37 Hospital Alemão Oswaldo Cruz, São Paulo, SP, Brasil.; 38 Rede D’Or, Regional ABC, Santo André, SP, Brasil.; 39 Hospital de Base de Bauru, Bauru, SP, Brasil.; 40 Secretaria Estadual de Saúde do Espírito Santo, Vitória, ES, Brasil.; 41 Universidade Federal do Espírito Santo, Hospital Universitário Cassiano Antonio Moraes, Vitória, ES, Brasil.; 42 Santa Casa de Misericórdia de Passos, Passos, MG, Brasil.; 43 Universidade Federal do Rio de Janeiro, Hospital Universitário Clementino Fraga Filho, Rio de Janeiro, RJ, Brasil.; 44 Universidade Federal do Rio de Janeiro, Rio de Janeiro, RJ, Brasil.; 45 Universidade Federal de Santa Catarina, Florianópolis, SC, Brasil.; 46 Universidade Federal da Bahia, Salvador, BA, Brasil.; 47 DaVita Tratamento Renal, Rio de Janeiro, RJ, Brasil.

**Keywords:** Hepatite C, Hemodiálise, Diálise Peritoneal, Insuficiência Renal Crônica, Antivirais, Resposta Viral Sustentada

## Abstract

**Introdução:**

A infecção pelo vírus da hepatite C (HCV) é um desafio de saúde pública, especialmente em pacientes em diálise, que apresentam maior vulnerabilidade e risco de transmissão. A estratégia de microeliminação em subgrupos de alto risco é fundamental para atingir as metas globais da Organização Mundial da Saúde.

**Métodos:**

Trata-se de um estudo nacional de implementação, multicêntrico e prospectivo, fundamentado no Registro Brasileiro para Eliminação da Hepatite C em Unidades de Diálise. As 904 unidades de diálise ativas do país foram convidadas; 384 (42,5%) aderiram voluntariamente e, em cada uma, todos os pacientes elegíveis foram arrolados entre 2021 e 2024. Avaliou-se a cascata de cuidado: rastreamento sorológico (anti-HCV), confirmação virológica (RNA-HCV), tratamento com antivirais de ação direta (DAAs) e resposta virológica sustentada (SVR).

**Resultados:**

Foram avaliados 66.601 pacientes, com prevalência nacional de anti-HCV de 2,08%. A cobertura de testagem para RNA-HCV foi de 65,4%, com positividade de 64,2% entre os testados, resultando em uma prevalência ajustada de viremia de 1,34%. Dos pacientes com viremia detectada, 74,2% iniciaram o tratamento, 92,6% completaram o esquema e 93,3% alcançaram a SVR. As principais barreiras incluíram indisponibilidade de exames e óbito.

**Discussão:**

Os resultados demonstram que o Brasil possui indicadores robustos de eficácia terapêutica, situando o país em estágio avançado para a eliminação do HCV na população dialítica. No entanto, persistem gargalos operacionais na confirmação diagnóstica.

**Conclusão:**

A eliminação do HCV em unidades de diálise é uma meta factível no Brasil. O sucesso depende da manutenção do rastreio universal, do tratamento imediato dos casos confirmados e da integração contínua entre a nefrologia e a hepatologia.

## INTRODUÇÃO

A infecção pelo vírus da hepatite C (HCV) permanece como um dos principais desafios de saúde pública global, apesar dos avanços terapêuticos obtidos com os antivirais de ação direta (DAAs), cujas taxas de cura são superiores a 95%. Estima-se que aproximadamente 58 milhões de pessoas vivam com infecção crônica pelo HCV e que ocorram 1,5 milhão de novos casos por ano no mundo^1,2^. Segundo a Organização Mundial da Saúde (OMS) e diretrizes internacionais, como as do *Kidney Disease: Improving Global Outcomes (KDIGO)* e da *European Association for the Study of the Liver (EASL)*, a eliminação da hepatite C até 2030 depende de estratégias integradas de rastreamento universal, confirmação diagnóstica, acesso oportuno ao tratamento e monitoramento da resposta virológica após o tratamento.

Neste sentido, o conceito de microeliminação – abordagem que se concentra no tratamento direcionado de subgrupos de alto risco – tem se mostrado uma estratégia eficaz para alcançar a meta global de eliminação do HCV. Entre os grupos de risco, destacam-se os pacientes em terapia renal substitutiva, particularmente aqueles em hemodiálise. Nessa população, a vulnerabilidade decorre de múltiplos fatores: manipulação frequente do acesso vascular, ambiente coletivo, exposição potencial a sangue e hemoderivados e a própria condição imunológica alterada. Além disso, surtos de transmissão nos serviços de diálise continuam a ser relatados, geralmente relacionados a falhas nas medidas de biossegurança. A consequência clínica é expressiva: indivíduos em diálise infectados pelo HCV apresentam pior qualidade de vida, maior taxa de hospitalização, maior mortalidade e risco aumentado de evolução para cirrose e carcinoma hepatocelular^3,4,5^.

Experiências internacionais demonstram que a eliminação do HCV em unidades de diálise é uma meta viável. O estudo multicêntrico FORMOSA, realizado em Taiwan, mostrou que a implementação de triagem universal associada ao tratamento com DAAs levou à redução da taxa de viremia de 7,7% em 2019 para apenas 0,6% em 2021. Esses resultados sustentam o conceito de “tratamento como prevenção”, ou seja, tratar todos os pacientes com infecção ativa como estratégia de microeliminação e prevenção de novos casos dentro das unidades de diálise^6^. De acordo com o Censo Brasileiro de Diálise da Sociedade Brasileira de Nefrologia, a prevalência de anticorpos anti-HCV (anti-HCV) em unidades de diálise apresentou queda significativa nas últimas duas décadas, passando de 19,3% em 2000 para 2,1% em 2023 – o que corresponde a cerca de 3,3 mil pacientes^7^. Ainda assim, essa taxa permanece três vezes superior à prevalência encontrada na população geral brasileira (0,7%). Uma revisão sistemática com metanálise de Niquini et al.^8^ documentou esse declínio no Brasil, ressalvando que estimativas agregadas de prevalência de HCV em hemodiálise são sensíveis ao tamanho dos estudos e a vieses de seleção e de publicação. Vale ressaltar que a presença de um resultado anti-HCV positivo indica contato prévio com o vírus; entretanto, ainda não há dados nacionais que mostrem a proporção desses indivíduos com infecção ativa, ou seja, com carga viral detectável.

A eliminação do HCV em pacientes em diálise no Brasil é uma meta factível, sobretudo pela disponibilidade universal dos DAAs no Sistema Único de Saúde (SUS). Para alcançá-la, é necessário fortalecer três pilares: (1) diagnóstico precoce e rastreamento sistemático; (2) tratamento universal e imediato dos casos detectados; e (3) prevenção rigorosa da transmissão nas unidades de diálise. A integração entre nefrologistas, hepatologistas, sociedades médicas e gestores públicos será essencial para alcançar os objetivos propostos pela Organização Mundial da Saúde (OMS), que estabelece como metas reduzir em 80% a incidência de novas infecções e em 65% a mortalidade relacionada ao HCV até 2030^3,9^. Nesse contexto, o Registro Brasileiro para Eliminação da Hepatite C nas Unidades de Diálise foi desenvolvido como uma iniciativa multicêntrica e colaborativa organizada pela Sociedade Brasileira de Nefrologia (SBN), em parceria com a Sociedade Brasileira de Hepatologia (SBH) e o Instituto Brasileiro do Fígado (IBRAFIG), com o intuito de identificar, tratar e monitorar a resposta virológica em pessoas sob tratamento dialítico no Brasil^10^.

## MÉTODOS

### Desenho do estudo

Este foi um estudo observacional, multicêntrico, prospectivo, baseado em registro, utilizando dados de mundo real provenientes de unidades de diálise participantes do Registro Brasileiro para Eliminação da Hepatite C nas Unidades de Diálise. O Registro foi concebido como uma iniciativa de saúde pública para rastrear, diagnosticar, tratar e monitorar a resposta virológica de pacientes com hepatite C crônica em diálise, bem como para identificar barreiras e oportunidades ao longo da linha de cuidado. O projeto foi conduzido pela Sociedade Brasileira de Nefrologia (SBN), em parceria com a Sociedade Brasileira de Hepatologia (SBH) e o Instituto Brasileiro do Fígado (IBRAFIG). A SBN, a SBH e o IBRAFIG nomearam, em cada unidade federativa, representantes estaduais, sendo um nefrologista e um hepatologista, responsáveis por convidar as unidades de diálise do estado, oferecer capacitação e acompanhar a coleta de dados. Todas as unidades participantes tiveram acesso a um curso de capacitação sobre rastreamento, diagnóstico e tratamento da hepatite C em diálise, segundo o Protocolo Clínico e Diretrizes Terapêuticas do Ministério da Saúde^9^.

### População

Foram convidadas a participar todas as 904 unidades de diálise ativas e cadastradas no Brasil. A adesão foi voluntária e condicionada à concordância com o protocolo padronizado de rastreamento, confirmação diagnóstica e tratamento, em conformidade com o protocolo clínico do Ministério da Saúde^9^. O convite foi universal e, em cada unidade aderente, todos os pacientes elegíveis foram arrolados sem subamostragem.

Foram elegíveis todos os pacientes em hemodiálise ou diálise peritoneal, independentemente do tempo em diálise ou da etiologia da doença renal crônica, atendidos nas unidades participantes durante o período do estudo. Em cada unidade aderente, todos os pacientes elegíveis foram convidados a participar e a assinar o Termo de Consentimento Livre e Esclarecido. Apenas aqueles que recusaram a participação foram excluídos.

### Procedimentos e fontes de dados

O cuidado foi estruturado em quatro etapas sequenciais, segundo o Protocolo Clínico e Diretrizes Terapêuticas para Hepatite C do Ministério da Saúde^9^: (1) rastreamento sorológico — identificação de pacientes com anti-HCV positivo, realizada como rotina das unidades de diálise; (2) confirmação virológica — realização do teste molecular de RNA-HCV em todos os pacientes com anti-HCV positivo; (3) tratamento antiviral — oferta e implementação de DAAs a todos os pacientes com RNA-HCV positivo; e (4) avaliação de SVR — monitoramento da carga viral ≥12 semanas após o término do tratamento.

A coleta de dados ocorreu entre março de 2021 e julho de 2024. A coleta seguiu duas fases. Na primeira, as unidades cadastraram-se junto à SBN e inseriram, na plataforma eletrônica do projeto (https://pesquisa.ibrafig.org.br/), os dados demográficos e clínicos dos pacientes, a modalidade de diálise e o resultado do anti-HCV. Na segunda fase, as informações foram consolidadas a partir de relatórios dos nefrologistas e hepatologistas designados em cada estado ou resgatadas pela equipe responsável pela gestão de dados, em planilhas padronizadas contendo: (1) resultado do RNA-HCV; (2) motivo de não realização do RNA-HCV; (3) início do tratamento; (4) conclusão do tratamento; (5) ocorrência de SVR; e (6) motivos de não tratamento entre os pacientes com RNA-HCV positivo.

### Desfechos e definições operacionais

A exposição prévia ao HCV foi definida como a presença de anticorpos anti-HCV detectáveis em teste sorológico. A viremia ativa foi definida como a presença de RNA-HCV detectável por transcrição reversa seguida de reação em cadeia da polimerase (RT-PCR). O tratamento completo foi considerado o término do regime com antivirais de ação direta (DAAs), conforme prescrição, com ou sem avaliação subsequente. A cura virológica, ou resposta virológica sustentada (SVR), foi definida como a ausência de RNA-HCV detectável ≥12 semanas após o término do tratamento.

O desfecho primário foi a prevalência de viremia ativa na população em diálise. Os desfechos secundários incluíram a prevalência de anti-HCV, a cobertura e a positividade do RNA-HCV, os indicadores da cascata de cuidado (início, conclusão do tratamento e SVR) e os motivos de não realização do RNA-HCV e de não tratamento.

### Análise estatística

As variáveis categóricas foram descritas como frequências absolutas e relativas e comparadas pelo teste do qui-quadrado de Pearson ou pelo teste exato de Fisher, quando ≥20% das células apresentaram frequência esperada <5. As variáveis contínuas foram descritas como mediana (intervalo interquartílico, IIQ), após avaliação da distribuição pelo teste de Shapiro–Wilk, e comparadas pelo teste de Mann–Whitney.

Foram analisadas as seguintes medidas: (1) número de participantes por estrato; (2) prevalência de anti-HCV (n/N); (3) cobertura do RNA-HCV entre os anti-HCV positivos (proporção que realizou o teste de RNA-HCV); (4) positividade do RNA-HCV entre os indivíduos com resultado conhecido (RNA-HCV positivo dividido pela soma de positivos e negativos, excluindo-se os casos sem resultado); e (5) prevalência de viremia na população total, apresentada de duas formas complementares: (5a) confirmada (observada), definida como RNA-HCV positivo dividido pelo total de participantes; e (5b) ajustada para verificação incompleta sob a premissa de dados ausentes ao acaso (*missing at random*, MAR), estimada como o produto entre a prevalência de anti-HCV e a positividade do RNA-HCV entre os testados. Descreveram-se, ainda, (6) a cascata de cuidado (anti-HCV positivo, RNA-HCV realizado, RNA-HCV positivo, tratados, tratamento completo e SVR), com as proporções de cada transição; (7) os motivos de não realização do RNA-HCV entre os anti-HCV positivos; e (8) os motivos de não tratamento entre os RNA-HCV positivos. A ausência de resultado em qualquer etapa foi tratada como desfecho de interesse e quantificada de forma explícita (ver análise estatística), em vez de motivar a exclusão.

As prevalências (anti-HCV e viremia confirmada), a cobertura e a positividade do RNA-HCV foram acompanhadas de intervalos de confiança binomiais de Wilson; a viremia ajustada (MAR) teve seu intervalo estimado pelo método delta para o produto de duas proporções independentes. Optou-se pelo intervalo de Wilson, em vez do intervalo de Wald, por seu melhor desempenho de cobertura em proporções próximas de zero, situação frequente nas estimativas estaduais de viremia. A variância foi obtida por Var(p_1_ · p_2_) ≈ p_2_^2^ · Var(p_1_) + p_1_^2^ · Var(p_2_). Para minimizar a ambiguidade de denominadores, a positividade do RNA-HCV considerou apenas indivíduos com resultado conhecido; a cobertura teve como denominador o total de anti-HCV positivos; e, na cascata, cada proporção foi calculada em relação ao passo imediatamente anterior (tratados entre RNA-HCV positivos, completos entre tratados e SVR entre completos). Em razão do grande tamanho amostral, as estimativas nacionais foram reportadas com intervalos de confiança de 99%, e as estimativas por estrato exibem os denominadores brutos (n/N), permitindo avaliar a precisão onde o n é pequeno. Todas as análises foram conduzidas no conjunto nacional e replicadas por região e por unidade federativa.

Para abordar a verificação incompleta do RNA-HCV, adotaram-se três estratégias complementares, sem exclusão de casos. Primeiro, a prevalência de viremia foi reportada de forma confirmada (limite inferior, que assume implicitamente que todos os não testados seriam negativos) e ajustada sob a premissa MAR (que assume que a positividade entre os não testados é igual à observada entre os testados, condicionalmente ao estrato). Segundo, realizou-se análise de sensibilidade por cenários extremos (*tipping-point*), atribuindo resultado negativo ou positivo a todos os não testados, de modo a delimitar os limites determinísticos da prevalência. Terceiro, para avaliar a plausibilidade da premissa MAR, compararam-se as características dos pacientes anti-HCV positivos com e sem resultado de RNA-HCV (idade, sexo, modalidade de diálise e etiologia da doença renal crônica) e testou-se a homogeneidade da cobertura do RNA-HCV entre as regiões, distinguindo a magnitude dos dados ausentes de seu eventual caráter informativo. Não se empregou imputação múltipla do *status* de RNA-HCV, por se tratar do próprio desfecho de confirmação, cuja imputação dependeria de um modelo não verificável.

Os valores de *p* são bicaudais, com α = 0,05, interpretados em conjunto com as estimativas e seus intervalos de confiança, e não como limiar dicotômico de decisão. As análises foram realizadas nos programas Jamovi 2.6.45 e R 4.3.1.

### Considerações éticas e aprovação do comitê de ética em pesquisa

O projeto foi aprovado pelo Comitê de Ética em Pesquisa (CEP) da Universidade Federal de São Paulo (CEP/UNIFESP), sob o parecer consubstanciado nº 4.412.987 (CAAE 39200420.2.1001.5505), emitido em 21 de novembro de 2020.

Todos os participantes assinaram o Termo de Consentimento Livre e Esclarecido (TCLE), em conformidade com a Resolução CNS nº 466/12. O estudo seguiu os princípios éticos da Declaração de Helsinque e as normas vigentes do Conselho Nacional de Saúde.

## RESULTADOS

Foram avaliadas 66.601 pessoas em diálise (63.028 em hemodiálise e 3.573 em diálise peritoneal), provenientes de 384 serviços em 26 estados brasileiros. Esses 384 serviços corresponderam a 42,5% das 904 unidades de diálise ativas convidadas. A participação distribuiu-se de forma desigual pelo território: Norte, com 27 centros (7,0%) e 3.609 pacientes; Nordeste, com 98 (25,5%) e 20.768; Centro-Oeste, com 66 (17,2%) e 8.983; Sudeste, com 128 (33,3%) e 24.367; e Sul, com 65 (16,9%) e 8.874. A distribuição dos participantes em relação à rede nacional de diálise é apresentada na Figura S1. A prevalência nacional de anti-HCV positivo foi de 2,08% (1.385/66.601; IC 99% 1,94–2,23). Ao estratificar por modalidade, observou-se prevalência de 2,17% em HD (1.366/63.028; IC 99% 2,02–2,32) e de 0,53% em DP (19/3.573; IC 99% 0,30–0,95). No plano regional, as estimativas de anti-HCV positivo indicaram heterogeneidade geográfica: Norte 2,83% (IC 99% 2,20–3,63), Nordeste 1,82% (IC 99% 1,60–2,08), Centro-Oeste 1,39% (IC 99% 1,11–1,75), Sudeste 2,47% (IC 99% 2,22–2,74) e Sul 2,01% (IC 99% 1,66–2,43). As estimativas por UF, com seus IC 99%, encontram-se detalhadas na Tabela 1 e na Figura 1A.

**Tabela 1:** Prevalência de positividade para anti-HCV e viremia (confirmada e ajustada), cobertura e positividade do RNA, por regiões e estados.

Local	N	Anti-HCV+, n	Prev. Anti-HCV+, % (IC 99%)	Cobertura de RNA, n/N, (%)	% de RNA+ entre os testados, (n/N)	Prev. confirmada de RNA+, % (IC 99%)	Prev. ajustada de RNA+ (MAR), % (IC 99%)
Brasil	66.601	1.385	2,1 (1,94–2,23)	906/1.385 (65,4)	64,2 (582/906)	0,9 (0,79–0,97)	1,3 (1,21–1,46)
Norte	3.609	102	2,8 (2,20–3,63)	48/102 (47,1)	43,8 (21/48)	0,6 (0,33–1,01)	1,2 (0,63–1,84)
Nordeste	20.768	379	1,8 (1,60–2,08)	239/379 (63,1)	56,5 (135/239)	0,7 (0,52–0,81)	1,0 (0,83–1,23)
Centro-Oeste	8.983	125	1,4 (1,11–1,75)	72/125 (57,6)	65,3 (47/72)	0,5 (0,36–0,76)	0,9 (0,62–1,20)
Sudeste	24.367	601	2,5 (2,22–2,74)	424/601 (70,5)	72,2 (306/424)	1,3 (1,08–1,45)	1,8 (1,55–2,01)
Sul	8.874	178	2,0 (1,66–2,43)	123/178 (69,1)	59,3 (73/123)	0,8 (0,61–1,11)	1,2 (0,87–1,51)
AC	452	38	8,4 (5,62–12,40)	3/38 (7,9)	66,7 (2/3)	0,4 (0,09–2,23)	5,6 (0,00–11,91)
AM	1.371	30	2,2 (1,38–3,46)	26/30 (86,7)	38,5 (10/26)	0,7 (0,33–1,60)	0,8 (0,18–1,51)
AP	361	6	1,7 (0,61–4,46)	4/6 (66,7)	50 (2/4)	0,6 (0,11–2,78)	0,8 (0,00–2,21)
PA	982	23	2,3 (1,38–3,94)	13/23 (56,5)	38,5 (5/13)	0,5 (0,17–1,51)	0,9 (0,00–1,84)
TO	90	1	1,1 (0,13–8,81)	1/1 (100)	100 (1/1)	1,1 (0,13–8,81)	1,1 (0,00–3,96)
RO	353	4	1,1 (0,34–3,73)	1/4 (25)	100 (1/1)	0,3 (0,03–2,37)	1,1 (0,00–2,58)
AL	1.991	43	2,2 (1,47–3,17)	38/43 (88,4)	13,2 (5/38)	0,3 (0,08–0,75)	0,3 (0,00–0,61)
BA	5.996	103	1,7 (1,34–2,21)	44/103 (42,7)	72,7 (32/44)	0,5 (0,34–0,84)	1,2 (0,82–1,68)
CE	3.177	53	1,7 (1,18–2,36)	36/53 (67,9)	52,8 (19/36)	0,6 (0,33–1,07)	0,9 (0,41–1,35)
MA	2.510	64	2,5 (1,86–3,49)	46/64 (71,9)	71,7 (33/46)	1,3 (0,84–2,04)	1,8 (1,10–2,56)
PB	1.147	37	3,2 (2,13–4,86)	27/37 (73)	51,9 (14/27)	1,2 (0,62–2,38)	1,7 (0,61–2,73)
PE	3.234	64	2,0 (1,44–2,72)	43/64 (67,2)	67,4 (29/43)	0,9 (0,56–1,44)	1,3 (0,77–1,89)
PI	549	6	1,1 (0,40–2,95)	0/6 (0)	—	—	—
RN	1.239	6	0,5 (0,18–1,32)	3/6 (50)	100 (3/3)	0,2 (0,06–0,95)	0,5 (0,00–0,99)
SE	925	3	0,3 (0,08–1,27)	2/3 (66,7)	0 (0/2)	—	—
DF	2.765	23	0,8 (0,49–1,41)	12/23 (52,2)	58,3 (7/12)	0,3 (0,10–0,65)	0,5 (0,08–0,89)
GO	3.118	52	1,7 (1,17–2,37)	39/52 (75)	59 (23/39)	0,7 (0,43–1,25)	1,0 (0,50–1,47)
MS	1.344	14	1,0 (0,53–2,03)	2/14 (14,3)	100 (2/2)	0,1 (0,03–0,76)	1,0 (0,33–1,76)
MT	1.756	36	2,1 (1,34–3,12)	19/36 (52,8)	78,9 (15/19)	0,9 (0,45–1,63)	1,6 (0,77–2,47)
ES	1.997	33	1,7 (1,06–2,56)	10/33 (30,3)	60 (6/10)	0,3 (0,11–0,82)	1,0 (0,20–1,78)
MG	7.750	114	1,5 (1,16–1,87)	77/114 (67,5)	42,9 (33/77)	0,4 (0,27–0,66)	0,6 (0,37–0,89)
RJ	3.816	126	3,3 (2,63–4,13)	88/126 (69,8)	68,2 (60/88)	1,6 (1,13–2,18)	2,3 (1,59–2,91)
SP	10.804	328	3,0 (2,64–3,49)	249/328 (75,9)	83,1 (207/249)	1,9 (1,60–2,29)	2,5 (2,12–2,92)
PR	3.455	44	1,3 (0,87–1,87)	22/44 (50)	72,7 (16/22)	0,5 (0,25–0,87)	0,9 (0,45–1,40)
RS	2.014	57	2,8 (2,02–3,95)	51/57 (89,5)	45,1 (23/51)	1,1 (0,67–1,93)	1,3 (0,61–1,94)
SC	3.405	77	2,3 (1,69–3,02)	50/77 (64,9)	68 (34/50)	1,0 (0,65–1,54)	1,5 (0,95–2,13)

Abreviaturas – AC: Acre; AM: Amazonas; AP: Amapá; PA: Pará; RO: Rondônia; TO: Tocantins; AL: Alagoas; BA: Bahia; CE: Ceará; MA: Maranhão; PB: Paraíba; PE: Pernambuco; PI: Piauí; RN: Rio Grande do Norte; SE: Sergipe; DF: Distrito Federal; GO: Goiás; MS: Mato Grosso do Sul; MT: Mato Grosso; ES: Espírito Santo; MG: Minas Gerais; RJ: Rio de Janeiro; SP: São Paulo; PR: Paraná; RS: Rio Grande do Sul; SC: Santa Catarina.

**Figura 1:** Prevalências observadas em cada unidade federativa: (A) Prevalência de exposição ao HCV (pacientes com anti-HCV positivo); (B) Porcentagem de pacientes com Anti-HCV positivo que apresentaram RNA-HCV positivo; (C) Prevalência de infecção ativa pelo HCV confirmada; (D) Prevalência de infecção ativa pelo HCV ajustada.

Entre os anti-HCV positivos, 66% eram do sexo masculino, com mediana de idade de 61 anos (IIQ 52–68). A etiologia predominante da doença renal crônica foi a hipertensão arterial sistêmica (40,8%), seguida por diabetes mellitus (21,8%) e glomerulonefrites crônicas (13,4%). Coinfecção viral foi identificada em 2,4% (34/1.385) dos pacientes, sendo 1,9% (27/1.385) por vírus da imunodeficiência humana e 0,5% (7/1.385) pelo vírus da hepatite B. Os dados da população anti-HCV positiva, por região e UF, encontram-se disponíveis na Tabela 2.

**Tabela 2:** Características clínicas dos pacientes anti-HCV positivos que realizaram o teste de RNA-HCV, comparando pacientes RNA-HCV positivos versus RNA-HCV negativos.

Característica	RNA-HCV+	RNA-HCV−	p
**n (grupo)**	582	324	—
**Idade, mediana [IIQ]**	60 [52–68]	61 [52–69]	0,7903
Sexo			0,5501
Feminino	191 (32,8%)	115 (35,5%)	
Masculino	390 (67,0%)	209 (64,5%)	
Não informado	1 (0,2%)	0 (0,0%)	
**Etiologia da DRC**			0,2687
HAS	245 (42,1%)	123 (38,0%)	
Diabetes mellitus	112 (19,2%)	79 (24,4%)	
Glomerulonefrite crônica	92 (15,8%)	45 (13,9%)	
DRPAD	14 (2,4%)	13 (4,0%)	
Outro	103 (17,7%)	57 (17,6%)	
Não informado	16 (2,7%)	7 (2,2%)	
**Tipo de diálise**			0,5295
Hemodiálise	571 (98,1%)	321 (99,1%)	
Diálise peritoneal	11 (1,9%)	3 (0,9%)	
**Presença de coinfecção**			0,9982
Presente	14 (2,4%)	8 (2,5%)	
Ausente	568 (97,6%)	316 (97,5%)	
**TGP (U/L), mediana [IIQ]**	14 [10–21]	13 [10–20]	0,0937

Abreviaturas – DRC: doença renal crônica; DRPAD: doença renal policística autossômica dominante do adulto; HAS: hipertensão arterial sistêmica; IIQ: intervalo interquartílico; RNA-HCV+: RNA do HCV detectável (viremia presente); RNA-HCV−: RNA do HCV indetectável; TGP: transaminase glutâmico-pirúvica (alanina aminotransferase, ALT); U/L: unidades por litro; *p*: valor de *p* (teste de Mann–Whitney para variáveis contínuas; teste do qui-quadrado de Pearson ou teste exato de Fisher para variáveis categóricas).

Entre os 1.385 indivíduos anti-HCV positivos, 906 realizaram o RNA-HCV, correspondendo a uma cobertura de 65,4%. Considerando apenas os anti-HCV positivos com resultado conhecido, a positividade do RNA-HCV foi de 64,2% (582/906). A cobertura de RNA variou substancialmente entre regiões, situando-se em 47,1% (48/102) no Norte, 63,1% (239/379) no Nordeste, 57,6% (72/125) no Centro-Oeste, 70,5% (424/601) no Sudeste e 69,1% (123/178) no Sul. Os dados por UF, com seus IC 99%, encontram-se detalhados na Tabela 1 e na Figura 1B. A diferença de cobertura entre as regiões foi estatisticamente significativa (χ^2^ = 27,6; gl = 4; *p* <0,001) (Figura S2). Os 479 indivíduos anti-HCV positivos sem resultado de RNA-HCV não diferiram dos 906 testados quanto à idade (mediana 61 vs. 60 anos; *p* = 0,70), ao sexo (*p* = 0,91) ou à modalidade de diálise (99,0% vs. 98,5% em hemodiálise; *p* = 0,60), havendo diferença apenas na distribuição da etiologia da doença renal crônica (*p* = 0,001).

A positividade no RNA-HCV foi observada em 582 de 66.601 participantes, o que corresponde a uma prevalência (observada) de 0,87% (IC 99% 0,79–0,97) (Figura 1C). Como nem todos os indivíduos anti-HCV positivos realizaram o RNA-HCV, calculou-se, também, uma prevalência ajustada. Nessa abordagem, a viremia nacional foi de 1,34% (IC 99% 1,21–1,46) (Figura 1D). A análise de sensibilidade por cenários extremos balizou a faixa plausível entre 0,87% (assumindo que todos os pacientes anti-HCV positivos não testados são RNA-HCV negativos) e 1,59% (assumindo que todos são RNA-HCV positivos). No recorte regional, as estimativas confirmadas de viremia foram de 0,58% no Norte (IC 99% 0,33–1,01), 0,65% no Nordeste (IC 99% 0,52–0,81), 0,52% no Centro-Oeste (IC 99% 0,36–0,76), 1,26% no Sudeste (IC 99% 1,08–1,45) e 0,82% no Sul (IC 99% 0,61–1,11). Após ajuste MAR, observaram-se 1,24% no Norte (IC 99% 0,63–1,84), 1,03% no Nordeste (IC 99% 0,83–1,23), 0,91% no Centro-Oeste (IC 99% 0,62–1,20), 1,78% no Sudeste (IC 99% 1,55–2,01) e 1,19% no Sul (IC 99% 0,87–1,51). Os dados por UF, com seus IC 99%, encontram-se detalhados na Tabela 1. Nos pacientes com RNA-HCV positivo, a informação de genótipo esteve disponível para 16,3% (95/582). A maioria apresentou níveis de ALT normais ou discretamente elevados, com tendência à maior mediana no grupo RNA-HCV positivo (14 vs. 13 U/L), porém sem significância estatística (*p* = 0,09) (Tabela 2).

A cascata de cuidado nacional (Figura 2) mostrou que, dos 582 pacientes com viremia detectada, 432 iniciaram tratamento, o que corresponde a 74,2% dos RNA-HCV positivos. Entre os tratados, 400 completaram o esquema, representando 92,6% de conclusão terapêutica. Dentre os que completaram o tratamento, a resposta virológica sustentada foi documentada em 373 indivíduos, equivalente a 93,3%. Os dados por região e por UF encontram-se detalhados na Tabela 3.

**Figura 2:** Cascata de cuidado do Registro Brasileiro para Eliminação da Hepatite C nas Unidades de Diálise.

**Tabela 3:** Cascata de cuidado entre pacientes RNA-HCV positivos: Brasil, regiões e unidades federativas.

Estrato	Tratados n*/N (%)	Tratamento completo n/N (%)	SVR n/N (%)
BRASIL	432/582 (74,2%)	400/432 (92,6%)	373/400 (93,3%)
NORTE	11/21 (52,4%)	10/11 (90,9%)	8/10 (80,0%)
NORDESTE	93/135 (68,9%)	84/93 (90,3%)	75/84 (89,3%)
CENTRO-OESTE	18/47 (38,3%)	16/18 (88,9%)	14/16 (87,5%)
SUDESTE	259/306 (84,6%)	240/259 (92,7%)	233/240 (97,1%)
SUL	51/73 (69,9%)	50/51 (98,0%)	43/50 (86,0%)
AC	2/2 (100,0%)	2/2 (100,0%)	2/2 (100,0%)
AM	3/10 (30,0%)	3/3 (100,0%)	3/3 (100,0%)
AP	2/2 (100,0%)	2/2 (100,0%)	0/2 (0,0%)
PA	2/5 (40,0%)	2/2 (100,0%)	2/2 (100,0%)
RO	1/1 (100,0%)	1/1 (100,0%)	1/1 (100,0%)
TO	1/1 (100,0%)	0/1 (0,0%)	—
AL	5/5 (100,0%)	5/5 (100,0%)	5/5 (100,0%)
BA	23/32 (71,9%)	18/23 (78,3%)	17/18 (94,4%)
CE	13/19 (68,4%)	11/13 (84,6%)	10/11 (90,9%)
MA	23/33 (69,7%)	23/23 (100,0%)	19/23 (82,6%)
PB	12/14 (85,7%)	12/12 (100,0%)	12/12 (100,0%)
PE	14/29 (48,3%)	12/14 (85,7%)	9/12 (75,0%)
PI	—	—	—
RN	3/3 (100,0%)	3/3 (100,0%)	3/3 (100,0%)
SE	—	—	—
DF	6/7 (85,7%)	6/6 (100,0%)	5/6 (83,3%)
GO	9/23 (39,1%)	7/9 (77,8%)	6/7 (85,7%)
MS	1/2 (50,0%)	1/1 (100,0%)	1/1 (100,0%)
MT	2/15 (13,3%)	2/2 (100,0%)	2/2 (100,0%)
ES	6/6 (100,0%)	6/6 (100,0%)	6/6 (100,0%)
MG	16/33 (48,5%)	16/16 (100,0%)	16/16 (100,0%)
RJ	50/60 (83,3%)	42/50 (84,0%)	35/42 (83,3%)
SP	187/207 (90,3%)	176/187 (94,1%)	176/176 (100,0%)
PR	14/16 (87,5%)	14/14 (100,0%)	14/14 (100,0%)
RS	19/23 (82,6%)	19/19 (100,0%)	12/19 (63,2%)
SC	18/34 (52,9%)	17/18 (94,4%)	17/17 (100,0%)

Abreviaturas – AC: Acre; AM: Amazonas; AP: Amapá; PA: Pará; RO: Rondônia; TO: Tocantins; AL: Alagoas; BA: Bahia; CE: Ceará; MA: Maranhão; PB: Paraíba; PE: Pernambuco; PI: Piauí; RN: Rio Grande do Norte; SE: Sergipe; DF: Distrito Federal; GO: Goiás; MS: Mato Grosso do Sul; MT: Mato Grosso; ES: Espírito Santo; MG: Minas Gerais; RJ: Rio de Janeiro; SP: São Paulo; PR: Paraná; RS: Rio Grande do Sul; SC: Santa Catarina.

Nota – *N: RNA-HCV+; SVR: resposta virológica sustentada.

A análise dos motivos de perdas elucidou gargalos distintos nas etapas de confirmação diagnóstica e de início terapêutico. Entre os 1.385 anti-HCV positivos, 459 não realizaram o RNA-HCV. As razões registradas foram: indisponibilidade do exame em 89 casos (19,4%), aguardo de avaliação com especialista em 36 (7,8%), óbito em 25 (5,4%), recusa ao exame em 21 (4,6%), ausência de condição clínica para se deslocar para a coleta do exame em 2 (0,4%) e perda de seguimento por transferência de unidade de diálise em 1 (0,2%), além de 285 registros como não informados (62,1%) (Figura 2). Além disso, a realização do RNA-HCV não foi informada em 20 indivíduos.

Entre os 582 pacientes com RNA-HCV positivo, 57 não iniciaram tratamento. As causas mais frequentes foram: recusa do paciente ao tratamento (24; 42,1%), óbito (15; 26,3%) e aguardo de avaliação de especialista (10; 17,5%), seguidas por ausência de condição clínica (3; 5,3%), falta de acesso ao medicamento (2; 3,5%), intolerância (1; 1,8%) e perda de seguimento por transplante renal (1; 1,8%) ou transferência de unidade de diálise (1; 1,8%) (Figura 2). O tratamento de 93 indivíduos não foi informado.

## DISCUSSÃO

A hepatite C permanece uma das principais causas de transplante hepático em diversos países e está associada a custos expressivos com internações e tratamento de complicações crônicas. Indivíduos infectados pelo vírus da hepatite C apresentam risco duas a três vezes maior de hospitalizações e mortalidade por causas hepáticas e extra-hepáticas, incluindo doenças cardiovasculares, renais e linfoproliferativas. Globalmente, regiões como o Leste Europeu e a Ásia Central ainda registram prevalência mais elevada, enquanto países da América do Norte e da Europa Ocidental vêm observando reduções significativas após a introdução dos DAAs e políticas agressivas de rastreamento e tratamento precoce, especialmente em populações de maior risco, como a de pacientes em diálise^1,2^.

No Brasil, o Censo Brasileiro de Diálise demonstra uma expressiva queda na prevalência de anticorpos anti-HCV, de 19,3% em 2000 para 2,1% em 2023. Contudo, o diagnóstico de infecção ativa – dependente da detecção da viremia por RNA-HCV – ainda é subutilizado na prática clínica. A ausência dessa confirmação laboratorial favorece a progressão da doença hepática no indivíduo infectado, o risco de soroconversão entre pacientes e a exposição ocupacional de profissionais de saúde^4,7^. Dados dos *Centers for Disease Control and Prevention* (CDC) indicam que mais da metade dos surtos de hepatite C entre 2008 e 2015 ocorreram em ambiente de hemodiálise, associados a múltiplos fatores: baixa adesão às ou violação das medidas de precaução universais, ausência de detecção precoce de casos suspeitos e atrasos no encaminhamento para tratamento. Soma-se a isso o fato de que não há vacinas disponíveis contra o vírus da hepatite C e não existe profilaxia após exposição. No Brasil, a contenção de um surto em uma unidade hospitalar de atenção ao rim crônico no Triângulo Mineiro ilustra a viabilidade da microeliminação local: busca ativa de casos, rastreamento das vias de transmissão, correção de técnicas de rotina e tratamento de todos os casos reativos interromperam a transmissão^11^.

Atualmente, a eliminação da hepatite C nas unidades de diálise é uma meta possível e requer rastreio universal e periódico, confirmação laboratorial da infecção ativa, início oportuno do tratamento e adesão rigorosa às medidas de biossegurança^4,12^. Revisões sistemáticas recentes indicam variação global entre 1% e 1,8% na população geral, dependendo do método, da região, do tamanho da amostra e do período analisado (pré- ou pós-DAAs)^13^. Em pacientes em diálise, a prevalência de anticorpos anti-HCV é significativamente mais elevada do que na população geral, refletindo o risco aumentado de transmissão associado à hemodiálise. Metanálises recentes demonstram prevalência média entre 5% e 10%, com ampla variabilidade regional e temporal. Países com protocolos rigorosos de controle de infecção e acesso ampliado à terapia antiviral têm registrado declínio acentuado dessas taxas^14,15,16^.

O presente registro multicêntrico representa o primeiro esforço coordenado em escala nacional para caracterizar a prevalência, a cascata de cuidado e as barreiras à eliminação do vírus da hepatite C (HCV) entre pacientes em diálise no Brasil. A inclusão de mais de 66.000 indivíduos provenientes de 384 unidades de diálise confere robustez estatística às estimativas e abrangência nacional ao panorama da linha de cuidado, refletindo a heterogeneidade regional e estrutural da rede de diálise brasileira.

Todos os indivíduos foram testados para anti-HCV com positividade de 2,1% (1.385 pessoas). A cobertura de RNA-HCV entre esses indivíduos foi de 65,4% e variou de 47% na região Norte a 70% na região Sudeste. As principais causas da ausência do resultado do exame em planilha foram: indisponibilidade de coleta do exame na própria unidade de diálise, dificuldade ou indisponibilidade de encaminhamento para o especialista, recusa à realização do exame ou simplesmente falta da informação em prontuário. Esses resultados traduzem gargalos operacionais na confirmação diagnóstica da infecção ativa, mas sinalizam oportunidades concretas de intervenção nos processos, tais como: incorporar rotinas de teste reflexo (anti-HCV positivo com reflexo automático para RNA-HCV), com coleta na própria unidade de diálise; fluxos de regulação simplificados para avaliação especializada; oferta de tratamento na própria unidade de diálise e medidas para fortalecer o engajamento da equipe de saúde.

Entre os indivíduos com anticorpos anti-HCV positivos, a proporção de pacientes com viremia detectável (RNA-HCV positivo) varia amplamente. Na população geral, aproximadamente 70% dos indivíduos desenvolverão infecção crônica, com RNA-HCV persistentemente positivo^3^. Em pacientes em diálise, essa proporção é altamente variável, oscilando entre 30% e 65% conforme o centro estudado^17^. Neste estudo, a positividade do RNA-HCV foi de 64,2% (582/906). Em termos programáticos, e assumindo que os não testados têm a mesma positividade dos testados, cerca de 6 em cada 10 pacientes dialíticos com anti-HCV positivo apresentaram infecção ativa (RNA-HCV positivo). Dessa forma, a testagem do RNA-HCV é essencial para confirmar a infecção ativa, conforme recomendam as principais diretrizes nacionais e internacionais^4,5,9^. A prevalência nacional de viremia entre indivíduos em diálise, de aproximadamente 1,34%, situa o Brasil em patamar semelhante ao de países que avançaram significativamente na microeliminação do HCV em diálise. Em Taiwan, Huang et al.^6^ demonstraram redução da viremia de 7,7% para 0,6% em apenas três anos de vigilância ativa, enquanto Hu et al.^18^ alcançaram cobertura diagnóstica de 98%, após implantação de um modelo colaborativo entre nefrologistas, hepatologistas e gestores públicos.

A cascata de cuidado foi robusta do ponto de vista clínico: 74,2% dos pacientes com viremia iniciaram tratamento (432/582), 92,6% completaram (400/432) e 93,3% alcançaram SVR (373/400). De fato, os antivirais de ação direta demonstraram elevada eficácia e segurança no tratamento da hepatite C em indivíduos em diálise, alcançando taxas de resposta virológica sustentada (SVR12 93–98%) na maioria das séries^4,5,19^. As perdas antes do início do tratamento com DAA foram explicadas principalmente por recusa (42,1%), óbito (26,3%) e aguardo de especialista (17,5%). Isso sugere dois eixos de resposta em saúde pública: (i) intervenções de adesão e educação (reduzir recusas, apoiar a decisão compartilhada) e (ii) acesso oportuno à avaliação/terapia (minimizar espera e desfechos fatais antes do início). A presença de óbito como motivo de não tratar reforça a necessidade de encurtar o tempo entre o diagnóstico e o início do DAA na diálise. Não houve diferenças estatisticamente significativas entre os grupos com e sem viremia quanto a sexo, idade, modalidade de diálise e ALT. O genótipo foi informado em apenas 16,3% daqueles com RNA-HCV positivo, o que limita inferências específicas.

Nossos resultados demonstram que a eliminação do HCV em populações dialíticas é viável quando há integração de cuidados e suporte institucional. No contexto brasileiro, as taxas de início terapêutico (74%) e de resposta virológica sustentada (SVR, 93%) entre os que completaram o tratamento reforçam a efetividade e a segurança dos DAAs. A microeliminação do HCV em unidades de diálise é possível. Para tal, fazem-se necessários rastreio universal periódico (anti-HCV positivo e confirmação por RNA-HCV), fluxo ágil para início do tratamento, monitorização dos indicadores: % testados, % RNA-positivos encaminhados, % que iniciaram DAA, SVR^14^, novos casos incidentes na unidade e integração da equipe multidisciplinar para a logística de tratamento e monitorização. A experiência de Taiwan^6,18^ nos ensina que a coordenação direta do sistema público de saúde, associada à formação de equipes multiprofissionais e à padronização do rastreio e do tratamento, permitiu atingir os indicadores de microeliminação recomendados pela OMS^3^.

O sucesso dos indicadores deste registro nacional está diretamente ligado ao modelo de saúde pública brasileiro. Desde a Constituição de 1988, a saúde é reconhecida como direito de todos e dever do Estado, possibilitando a organização do Sistema Único de Saúde (SUS) sob os princípios da universalidade e da equidade. No contexto da hepatite C, essa diretriz se concretiza na oferta gratuita de antivirais de ação direta pelo Ministério da Saúde à população dialítica em terapia renal substitutiva^9^. O fornecimento universal e ininterrupto dessas medicações de alto custo é essencial, pois remove barreiras financeiras críticas, viabiliza a microeliminação do HCV e reforça o compromisso institucional com a proteção de grupos vulneráveis.

No Brasil, o modelo liderado pela SBN em parceria com a SBH e o IBRAFIG segue o mesmo princípio, promovendo sinergia entre especialistas e gestores para atingir metas de eliminação nas unidades de diálise. Os resultados sugerem que o Brasil está em estágio avançado para alcançar a eliminação do HCV na população dialítica. Considerando a prevalência ajustada de viremia de 1,34% e a atual população estimada em diálise (cerca de 160 mil pessoas), o número de indivíduos com infecção ativa seria inferior a dois mil, contingente plenamente tratável dentro da capacidade operacional do SUS. O impacto esperado da eliminação viral inclui melhora da qualidade de vida, redução de hospitalizações e da mortalidade cardiovascular e hepática, além da eliminação da necessidade de isolamento de máquinas para pacientes infectados. O conceito de “tratamento como prevenção”, reforçado pelo estudo FORMOSA-LIKE^6^, destaca a importância do tratamento universal como ferramenta de prevenção coletiva. A erradicação do vírus dentro das unidades de diálise reduz não apenas o risco de reinfecção e de novos casos, mas também a carga de estigma associada à infecção.

A distribuição regional dos participantes reflete a adesão voluntária das unidades, e não a demografia populacional nem a distribuição nacional de pacientes em diálise. Frente à rede nacional, o Sudeste, que concentra cerca de 47% das unidades ativas do país, ficou sub-representado (33,3% dos centros e 36,6% dos pacientes do Registro), enquanto o Nordeste e o Centro-Oeste ficaram sobrerrepresentados, em razão da maior mobilização nessas regiões (Figura S1). Essa heterogeneidade de adesão explica, por exemplo, por que o número de participantes do Nordeste (20.768) supera o do Sul (8.874).

Este estudo apresenta limitações. Primeiro, embora o arrolamento dos pacientes dentro de cada unidade tenha sido censitário, a participação das unidades foi voluntária (384 de 904 unidades ativas; 42,5%), de modo que o conjunto de centros constitui uma amostra não probabilística de serviços, possivelmente sujeita a um viés de autosseleção, em que unidades com melhor estrutura ou maior engajamento das equipes tendem a aderir preferencialmente a iniciativas dessa natureza. As estimativas de prevalência devem, portanto, ser lidas como descritivas do conjunto arrolado, e não como parâmetros nacionais ponderados, recomendando-se cautela na extrapolação direta, sobretudo para regiões sub-representadas. Como observado por Niquini et al.^8^, estimativas de prevalência de HCV em diálise são particularmente sensíveis a vieses de seleção e de publicação. Segundo, a verificação do RNA-HCV foi incompleta (cobertura de 65,4%) e diferencial entre as regiões. Mitigamos essa limitação pela estimativa ajustada sob a premissa MAR, pela análise de sensibilidade que delimitou a viremia entre 0,87% e 1,59% (Figura S3) e pela demonstração de que os pacientes não testados foram semelhantes aos testados nas principais características observáveis (idade, sexo e modalidade), o que torna improvável, ainda que não exclua, um viés relevante por ausência não aleatória. Reconhecemos, contudo, que a premissa MAR não é diretamente testável. Terceiro, a informação de genótipo esteve disponível em apenas 16,3% dos casos com viremia, limitando inferências sobre a distribuição genotípica. Por fim, desfechos clínicos de longo prazo e a incidência de novos casos por unidade não foram coletados, o que deverá ser contemplado na transformação do Registro em sistema permanente de vigilância. Ressalte-se que os indicadores de efetividade terapêutica (início, conclusão e SVR) referem-se a pacientes efetivamente tratados, sendo, portanto, robustos à questão da representatividade dos centros.

Este estudo representa a primeira iniciativa nacional de microeliminação da hepatite C em pacientes em terapia dialítica, abrangendo todo um país de dimensões continentais e marcado por expressiva heterogeneidade regional no acesso, na infraestrutura diagnóstica e na organização do cuidado. A experiência brasileira demonstra que a eliminação da hepatite C em unidades de diálise é uma meta factível quando apoiada em três pilares: rastreio universal e periódico, tratamento imediato dos casos confirmados e adesão rigorosa às práticas de biossegurança. A cooperação entre sociedades médicas e gestores públicos, já consolidada por meio do Registro Brasileiro para Eliminação da Hepatite C nas Unidades de Diálise, constitui um modelo efetivo de integração entre a nefrologia e a hepatologia no enfrentamento de um problema de saúde pública persistente. A transformação desse registro em um sistema permanente de vigilância e monitoramento poderá consolidar a microeliminação do HCV e fortalecer a qualidade assistencial nas unidades de diálise. O Brasil dispõe dos recursos terapêuticos, da estrutura institucional e da capacidade técnica necessários para erradicar o HCV na população dialítica. O desafio remanescente é essencialmente operacional: assegurar que todo paciente seja testado, tratado e curado, e que cada unidade de diálise se torne, de fato, um ambiente livre da hepatite C.

## Data Availability

Os conjuntos de dados gerados e/ou analisados durante o presente estudo não estão disponíveis publicamente devido a restrições éticas e de privacidade, mas podem ser obtidos junto ao autor correspondente mediante solicitação razoável.

## MATERIAL SUPLEMENTAR

O seguinte material online está disponível para o presente artigo:

Figura S1 – Representatividade regional do Registro frente às unidades de diálise. (A) Distribuição percentual de centros participantes do Registro versus unidades de diálise ativas no Brasil (Censo Brasileiro de Diálise da SBN). (B) Distribuição percentual de pacientes incluídos no Registro versus distribuição nacional. As diferenças refletem a adesão voluntária das unidades, e não a demografia populacional.

Figura S2 – Cobertura do teste de RNA-HCV entre pacientes anti-HCV positivos, por região, com intervalos de confiança de 95% (método de Wilson). A linha tracejada indica a cobertura nacional (65,4%). A heterogeneidade entre regiões foi estatisticamente significativa (χ^2^ = 27,6; gl = 4; *p* < 0,001).

Figura S3 – Análise de sensibilidade da prevalência de viremia ativa (RNA-HCV+) na população dialítica. A estimativa confirmada (0,87%) corresponde ao limite inferior (todos os não testados assumidos como negativos) e o cenário máximo (1,59%), ao limite superior (todos positivos). A estimativa ajustada (MAR) de 1,34% situa-se na faixa plausível delimitada por esses extremos. As barras indicam IC de 99%.
